# Population genetics and microevolution of clinical *Candida glabrata* reveals recombinant sequence types and hyper-variation within mitochondrial genomes, virulence genes, and drug targets

**DOI:** 10.1093/genetics/iyac031

**Published:** 2022-02-23

**Authors:** Nicolas Helmstetter, Aleksandra D Chybowska, Christopher Delaney, Alessandra Da Silva Dantas, Hugh Gifford, Theresa Wacker, Carol Munro, Adilia Warris, Brian Jones, Christina A Cuomo, Duncan Wilson, Gordon Ramage, Rhys A Farrer

**Affiliations:** Medical Research Council, Centre for Medical Mycology, University of Exeter, Exeter EX4 4QD UK; Institute of Medical Sciences, University of Aberdeen, Aberdeen AB25 2ZD, UK; School of Medicine, College of Medical, Veterinary and Life Sciences, University of Glasgow, Glasgow G12 8QQ, UK; Medical Research Council, Centre for Medical Mycology, University of Exeter, Exeter EX4 4QD UK; Medical Research Council, Centre for Medical Mycology, University of Exeter, Exeter EX4 4QD UK; Medical Research Council, Centre for Medical Mycology, University of Exeter, Exeter EX4 4QD UK; Institute of Medical Sciences, University of Aberdeen, Aberdeen AB25 2ZD, UK; Medical Research Council, Centre for Medical Mycology, University of Exeter, Exeter EX4 4QD UK; Institute of Infection, Immunity & Inflammation, University of Glasgow, Glasgow G12 8TA, UK; Broad Institute of MIT and Harvard, Cambridge, MA 02142, USA; Medical Research Council, Centre for Medical Mycology, University of Exeter, Exeter EX4 4QD UK; School of Medicine, College of Medical, Veterinary and Life Sciences, University of Glasgow, Glasgow G12 8QQ, UK; Medical Research Council, Centre for Medical Mycology, University of Exeter, Exeter EX4 4QD UK; Broad Institute of MIT and Harvard, Cambridge, MA 02142, USA

**Keywords:** *Candida glabrata*, genome sequencing, epidemiology, candidiasis, microevolution, mitochondria, drug-resistance, evolution

## Abstract

*Candida glabrata* is the second most common etiological cause of worldwide systemic candidiasis in adult patients. Genome analysis of 68 isolates from 8 hospitals across Scotland, together with 83 global isolates, revealed insights into the population genetics and evolution of *C. glabrata*. Clinical isolates of *C. glabrata* from across Scotland are highly genetically diverse, including at least 19 separate sequence types that have been recovered previously in globally diverse locations, and 1 newly discovered sequence type. Several sequence types had evidence for ancestral recombination, suggesting transmission between distinct geographical regions has coincided with genetic exchange arising in new clades. Three isolates were missing MATα1, potentially representing a second mating type. Signatures of positive selection were identified in every sequence type including enrichment for epithelial adhesins thought to facilitate fungal adhesin to human epithelial cells. In patent microevolution was identified from 7 sets of recurrent cases of candidiasis, revealing an enrichment for nonsynonymous and frameshift indels in cell surface proteins. Microevolution within patients also affected epithelial adhesins genes, and several genes involved in drug resistance including the ergosterol synthesis gene *ERG4* and the echinocandin target *FKS1*/2, the latter coinciding with a marked drop in fluconazole minimum inhibitory concentration. In addition to nuclear genome diversity, the *C. glabrata* mitochondrial genome was particularly diverse, with reduced conserved sequence and conserved protein-encoding genes in all nonreference ST15 isolates. Together, this study highlights the genetic diversity within the *C. glabrata* population that may impact virulence and drug resistance, and 2 major mechanisms generating this diversity: microevolution and genetic exchange/recombination.

## Introduction 

*Candida* is the most prominent genus of the Debaryomycetaceae family, with over 400 genetically and phenotypically diverse species currently described (Daniel *et al.* 2014). Many of these species are harmless commensals of the mucous membranes and digestive tracts of healthy individuals. Approximately 30 *Candida* species are of clinical importance in humans. Most of these species that are capable of causing disease in humans belong to the CTG-Serine clade, including *Candida* *albicans*, *Candida* *dubliniensis*, *Candida* *tropicalis*, *Candida* *parapsilosis*, *Candida* *lusitaniae*, *Candida* *guilliermondii*, and *Candida* *auris*, while others such as *Candida* *glabrata* and *Candida* *bracarensis* belong to the genetically distant Nakaseomyces clade (Daniel *et al.* 2014; Turner and Butler 2014). In adult patients, *C. glabrata* is the second most commonly isolated species after *C. albicans*, which together cause approximately 3 quarters of all systemic candidiasis (Perlroth *et al.* 2007; Rajendran *et al.* 2016a). Infections caused by these species range from mild vulvovaginal candidiasis (VVC or thrush) to severe, drug resistant and difficult to treat invasive infections affecting single organs or the blood stream (candidemia) with or without dissemination to the heart, brain, kidneys, and other parts of the body (Mullick *et al.* 2006). Bloodstream infections caused by *Candida* spp. are associated with mortality rates of 30–60% (Hirano *et al.* 2015; Lamoth *et al.* 2018). Candidemia is associated with diverse risk factors including neutropenia, chemotherapy, diabetes, old age, compromised immune function, prolonged antibiotic and steroid treatment, and intravenous catheters that can harbor fungal biofilms (Yapar 2014). Pathogenic *Candida* species including *C. glabrata* have also exhibited alarming increases in resistance against all major classes of antifungal drugs, hindering effective treatments and resulting in increasing mortality rates (Cowen *et al.* 2002; Odds *et al.* 2007; Pfaller *et al.* 2012; Rajendran *et al.* 2016a; Barber *et al.* 2019).

*Candida* *glabrata* typically grows in the yeast form and is considered to have evolved an infection strategy based on stealth and evasion without causing severe damage in murine models (Brunke and Hube 2013). This ability of *C. glabrata* and some of its relatives in the Nakaseomyces clade to infect humans is thought to have evolved recently (Gabaldón *et al.* 2013), as several of its closest relatives have to date been exclusively isolated environmentally (*Caenorhabditis* *castellii*, *Nakaseomyces* *baccilisporus*, and *Nakaseomyces* *delphensis*) (Gabaldón *et al.* 2013). Pathogenicity in the Nakaseomyces correlates with the number of epithelial adhesins (EPA) encoded in their genomes, which facilitate adherence and colonization of human epithelial cells (Cormack *et al.* 1999). In contrast to *C. albicans*, the pathogenicity of Nakaseomyces species does not coincide with number or presence of Phospholipase-B and Superoxide Dismutase genes (Ibrahim *et al.* 1995; Martchenko *et al.* 2004; Cafarchia *et al.* 2008; Bink *et al.* 2011). Many *Candida* genes involved in virulence are therefore likely to have diverse functions, some of which may not be conserved between distant clades.

Like many fungal pathogens, *C. glabrata’*s niche(s) and life cycle are poorly understood*.* *Candida* *glabrata* is increasingly identified among clinical samples where it is responsible for an increasing proportion of cases of candidemia (Beck-Sagué and Jarvis 1993; Rajendran *et al.* 2016a). *Candida* *glabrata* has also been identified environmentally, including as a component of the mycobiota of yellow-legged gulls (Al-Yasiri *et al.* 2016), in droppings and cloaca swabs of birds of prey, migratory birds and passeriformes (Cafarchia *et al.* 2008), and other potentially transitory sources including spontaneously fermenting coffee beans (de Melo Pereira *et al.* 2014). Concerted efforts for sampling are required to determine the true ecological distribution of *C. glabrata*, as they are for several other important *Candida* species such as *C. auris* (Arora *et al.* 2021). Furthermore, the relatedness of global isolates and their routes of transmission (either patient to patient, or between patient and the environment) requires further studies comparing genotypes to collected metadata including the location of isolation.

High levels of genetic heterogeneity have been identified in the *C. glabrata* population, as molecular methods have identified diverse strains, clades and sequence types (STs) both inter- and intra-nationally (Biswas *et al.* 2018; Carreté *et al.* 2018). As of January 2022, the multi locus sequence type (MLST) database for *C. glabrata* included 233 STs from 1,414 isolates from 29 countries, based on the sequence identity for 6 genetic loci (Gabaldón *et al.* 2020). All isolates of *C. glabrata* reported to date have been haploid, with occasional aneuploids e.g. transitory disomies of chromosomes E and G (Carreté *et al.* 2018).

Genetic heterogeneity within many fungal populations is shaped by their ability to switch between clonal and sexual recombination (Drenth *et al.* 2019). The ability for *C. glabrata* to undergo a sexual cycle remains unknown, with all reported attempts in the laboratory to encourage mating thus far unsuccessful. *Candida* *glabrata* has therefore been regarded as an asexual species, despite the presence of well-conserved mating loci (Wong *et al.* 2003; Gabaldón and Fairhead 2019) and 14 examples of phylogenetic incompatibilities from multi locus sequencing (Dodgson *et al.* 2005). More recent genomic analysis from 34 globally isolated *C. glabrata* strains revealed evidence of population admixture, suggesting a thus far undiscovered sexual cycle (Carreté *et al.* 2018). Greater sampling efforts and genomic analyses are therefore required to fully explore signatures of adaptation, virulence, and recombination.

In this study, we explore the population genetics and microevolution of *C. glabrata* using comparative genomic analysis of 68 clinical *C. glabrata* isolates from 8 hospitals across Scotland, combined with 83 publicly available and globally isolated genomes, finding evidence of recombinant STs, hypervariable mitochondrial genomes, as well as variation in virulence genes and drug targets between STs and between serial isolates from prolonged or recurrent infection.

## Materials and methods

### Library preparation, sequencing, and antifungal tests

*Candida glabrata* was collected from blood in 2012 from 8 hospitals in Scotland (Supplementary Table 1). These isolates were collected as part of a retrospective study of all cases of *Candida* blood stream infections carried out within Scotland under NHS Caldicott Guardian approval from March 2012 to February 2013, as described previously (Rajendran *et al.* 2016a, 2016b).

Genomic and mitochondrial DNA was extracted from 68 isolates using the QIAamp DNA mini extraction kit (Qiagen) according to the manufacturer’s instructions. A small modification was made prior to extraction which was to mechanical disrupt the yeast. This was achieved by bead-beating the cells with sterile acid-washed 0.5 mm diameter glass beads (Thistle Scientific) for 3 × 30 s. Following isolation and extraction using the QIAamp columns the DNA was eluted into elution buffer before storage at −20°C and transport to the sequencing facility.

Library preparation was performed by the Centre for Genome-Enabled Biology and Medicine at the University of Aberdeen. Briefly, gDNA quality was assessed on a Tapestation 4200 with a high sensitivity genomic DNA tape (Agilent) and quantified by fluorimetry using Quant-IT dsDNA high-sensitivity (HS) assay (Thermo Fisher). Dual indexed Illumina libraries were prepared from 1 ng purified gDNA using an Illumina Nextera XT DNA library preparation kit and Nextera XT v2 indices, which were purified from free adapters using AMPure XP beads (Beckman Coulter). Libraries were quantified using Quant-IT dsDNA HS assay (Thermo Fisher) and average fragment size was calculated on Tapestation 4200 (Agilent), then equimolar pooled at 10 nM. Concentration of the pool was verified by qPCR (Kapa library quantification kit, Roche) on QuantStudio 6 using SYBR green, and 1.8 pM of the library pool was sequenced on an Illumina NextSeq500 with 150 bp paired-end reads and 8 bp index reads to average alignment depths of 41.9X (Supplementary Table 2). This data was supplemented with paired-end illumina reads from Carreté *et al.* (2018), and isolate CBS138 from Xu *et al.* (2020).

Minimum inhibitory concentration (MIC) tests for fluconazole were performed at the Mycology Reference Laboratory, Public Health England, Bristol, according to standard Clinical and Laboratory Standards Institute (CLSI) broth microdilution M27 guidelines (Alexander and Clinical and Laboratory Standards Institute 2017).

### Variant calling

The Genome Analysis Toolkit (GATK) v.4.1.2.0 (McKenna *et al.* 2010) was used to call variants. Our workflow description language (WDL) scripts were executed by Cromwell workflow execution engine v.48 (Voss *et al.* 2017). Briefly, raw sequences were pre-processed by mapping reads to the reference genome *C. glabrata* CBS138 using BWA-MEM v.0.7.17 (Li 2013). Next, duplicates were marked, and the resulting file was sorted by coordinate order. Intervals were created using a custom bash script allowing parallel analysis of large batches of genomics data. Using the scatter-gather approach, HaplotypeCaller was executed in GVCF mode with the haploid ploidy flag. Variants were imported to GATK 4 GenomicsDB and hard filtered if QualByDepth (QD) < 2.0, FisherStrand (FS) > 60.0, root mean square mapping quality (MQ) < 40.0, genotype quality (GQ) ≥50, allele depth (AD) ≥ 0.8, or Coverage (DP) ≥ 10.

To identify aneuploid chromosomes, depth of coverage was calculated for each of 206 fungal samples. Sorted BAM files prepared in the pre-processing phase of SNP calling were passed to Samtools v.1.2 (Li *et al.* 2009) and mpileup files were generated. Read depth was normalized by total alignment depth and plotted against the location in the genome using 10 kb nonoverlapping sliding windows. To identify structural variation, assembly de novo was achieved using Spades v3.12 (Bankevich *et al.* 2012) using default parameters.

### Phylogenetic and population genetic analysis

To construct species-specific phylogenetic trees, all sites that were either a homozygous reference or SNP in every isolate were identified using ECATools (https://github.com/rhysf/ECATools) and concatenated into a FASTA file. Our rooted tree *C. bracarensis* included 1,198 phylogenetically informative sites, while the unrooted *C. glabrata* tree included 34,980 phylogenetically informative sites. Phylogenetic trees were constructed with RAxML PThreads v.7.7.8 (Stamatakis 2006) using the general-time-reversible model and CAT rate approximation with 100 bootstrap support, both with rooting to *C. bracarensis* AGP (Correia *et al.* 2006) or midpoint rooting without *C. bracarensis*. We constructed a tree using the same models with 1000 bootstrap support for all Saccharomycetaceae species that had a genome assembly in NCBI or JGI Mycocosm. We also constructed neighbor-joining trees using PAUP v4.0b10 (Swofford n.d.) and a NeighborNet Network with SplitsTree v.4.15.1 (Huson 1998). Trees were visualized using FigTree v. 1.4.4 (http://tree.bio.ed.ac.uk/software/figtree/).

A multisample variant call format (VCF) corresponding to all 151 genomes was made with VCFTools v0.1.12 vcf-merge (Danecek *et al.* 2011) and converted to ped and map file formats for use in PLINK v1.90 (Purcell *et al.* 2007). VCFTools was used to calculate genetic diversity metric pi, using the –site-pi parameter. Unsupervised ADMIXTURE (Alexander *et al.* 2009) (settings: –haploid=“*” –s time) was run on a moderately linkage disequilibrium (LD)-pruned alignment (PLINK –indep-pairwise 60 10 0.1) for values of K between 1 and 35. A value of *K* = 20 provided the lowest cross-validation error. Principle component analysis was performed using SmartPCA v4 (Abraham and Inouye 2014). Consensus gene sequences for each isolate were generated, and genes *FKS2* (CAGL0K04037g) positions 240-828, *LEU2* (CAGL0H03795g), *NMT1* (CAGL0A04059g), *TRP1* (CAGL0C04092g), *UGP1* (CAGL0L01925g), and *URA3* (CAGL0I03080g) were used to identify known MLST for each of the isolates, and confirm that isolate CG57 was an unknown MLST and registered as the new ST204 on PubMLST (https://pubmlst.org/organisms/candida-glabrata [last accessed January 2022]) (Jolley *et al.* 2018).

We applied Weir’s estimator (Hudson and Kaplan 1985) of Wright’s Fixation Index (*F_ST_*) according to the equations given in Multilocus 1.3 (Agapow and Burt 2001) using nonoverlapping sliding windows. The scripts have been made available online (https://github.com/rhysf/FSTwindows).

### Selection and microevolutionary analysis

The direction and magnitude of natural selection for each ST were assessed by measuring the rates of nonsynonymous substitution (*dN*), synonymous substitution (*dS*), and omega (ω = *dN*/*dS*) using the yn00 program of PAML (Yang 2007), which implements the Yang and Nielsen method, taking into account codon bias (Yang and Nielsen 2000). Further GC corrections were not applied. The program was run on every gene in each isolate using the standard nuclear code translation table. To examine the functional significance of genes with ω  >  1, we evaluated their Pfam domains and gene ontology (GO) terms for statistical enrichment (genes with ω > 1 vs, the remaining genes) using the 2-tailed Fisher exact test with Storey false discovery rate (FDR)-corrected *P*-values (q) of < 0.05. GO terms were acquired using Blast2Go v6.0.1 (Conesa *et al.* 2005, p. 2) using Blastp-fast to the NCBI BLAST nr-database (*E*-value < 1E-5).

Genes of interest were defined including both *FKS* and 12 *ERG* pathway genes, as well as all genes listed in Table 1 of Weig *et al.* (2004), which included adhesins including *EPA* genes, aspartic proteases, phospholipases, cell wall biogenesis, structural wall proteins, regulatory, efflux pumps. This gene list was then screened for genes with either signature of positive selection or those undergoing microevolutionary changes (nonsynonymous and frameshift indels).

**Table 1:** GO-term and PFAM enrichment [2-tailed Fisher exact test with FDR-corrected *P*-values (q) of** **<** **0.05] for genes with *dN*/*dS* (ω) > 1, and genes with either microevolutionary frameshifts or nonsynonymous mutations across the 7 sets of serial isolates.

Category							
dN/dS > 1	GO/PFAM term	**Genes** ω **< 1**	**Genes** ω **> 1**	Fisher *P*	*Q*-value	Rel. prop	GO/PFAM description
	GO: 0003723	428	25	2.26E-04	3.04E-02	1.95	RNA binding
	GO: 0003735	152	3	7.38E-05	1.49E-02	5.78	Structural constituent of ribosome
	GO: 0003824	1745	155	8.61E-05	1.64E-02	1.28	Catalytic activity
	GO: 0005515	1100	81	4.48E-06	1.41E-03	1.55	Protein binding
	GO: 0005740	278	11	5.08E-05	1.06E-02	2.88	Mitochondrial envelope
	GO: 0005759	160	4	1.90E-04	2.65E-02	4.56	Mitochondrial matrix
	GO: 0006412	287	11	2.68E-05	6.05E-03	2.98	Translation
	GO: 0019693	100	1	3.63E-04	4.35E-02	11.4	Ribose phosphate metabolic process
	GO:0022626	87	0	1.16E-04	1.99E-02	N/A	Cytosolic ribosome
	GO:0022857	277	13	4.08E-04	4.70E-02	2.43	Transmembrane transporter activity
	GO:0036094	724	52	2.58E-04	3.31E-02	1.59	Small molecule binding
	GO:0043168	689	48	1.89E-04	2.65E-02	1.64	Anion binding
	GO:0044281	498	27	1.57E-05	3.86E-03	2.1	Small molecule metabolic process
	GO:0044391	143	3	2.32E-04	3.05E-02	5.44	Ribosomal subunit
	GO:0071840	1325	113	2.76E-04	3.47E-02	1.34	Cellular component organization or biogenesis
	GO:1901362	364	19	1.92E-04	2.65E-02	2.18	Organic cyclic compound biosynthetic process
	PF00624.20	4	23	6.53E-20	7.42E-17	0.02	Flocculin repeat
	PF10528.11	12	10	1.87E-05	1.07E-02	0.13	GLEYA domain
	PF00514.25	8	8	5.72E-05	2.17E-02	0.11	Armadillo repeat
**Microevolution (Frameshift)**	**GO/PFAM term**	**Genes without frameshift**	**Genes with frameshift**	**Fisher *P***	***Q*-value**	**Rel. prop**	**GO/PFAM description**
	GO:0009986	30	9	7.53E-12	4.26E-08	0.03	Cell surface
	GO:0009987	3616	17	1.20E-05	3.38E-02	1.7	Cellular process
	PF05001.15	0	17	1.10E-39	1.25E-36	0	RNA polymerase Rpb1 C-terminal repeat
	PF10528.11	13	9	5.54E-15	3.15E-12	0.01	GLEYA domain
	PF00399.21	24	9	4.09E-13	1.55E-10	0.02	Yeast PIR protein repeat
	PF08238.14	14	6	2.64E-09	7.49E-07	0.02	Sel1 repeat
	PF11765.10	6	3	2.58E-05	5.86E-03	0.01	Hyphally regulated cell wall protein N-terminal
	PF09770.11	0	2	4.79E-05	9.08E-03	0	Topoisomerase II-associated protein PAT1
**Microevolution (nonsynonymous)**	**GO/PFAM term**	**Genes without nonsynonymous**	**Genes with nonsynonymous**	**Fisher *P***	***Q*-value**	**Rel. prop**	**GO/PFAM description**
	GO:0009986	28	11	7.88E-10	4.45E-06	0.06	Cell surface
	PF10528.11	12	10	3.14E-11	3.58E-08	0.03	GLEYA domain
	PF11765.10	4	5	1.03E-06	5.87E-04	0.02	Hyphally regulated cell wall protein N-terminal

The relative proportion (Rel. prop) was calculated as (number of terms in set 1/number of terms in set 2) × (genes with any terms in set 2/genes with any terms in set 1).

## Results

### Recombinant sequence types in Scottish clinical samples

**Fig. 1.**
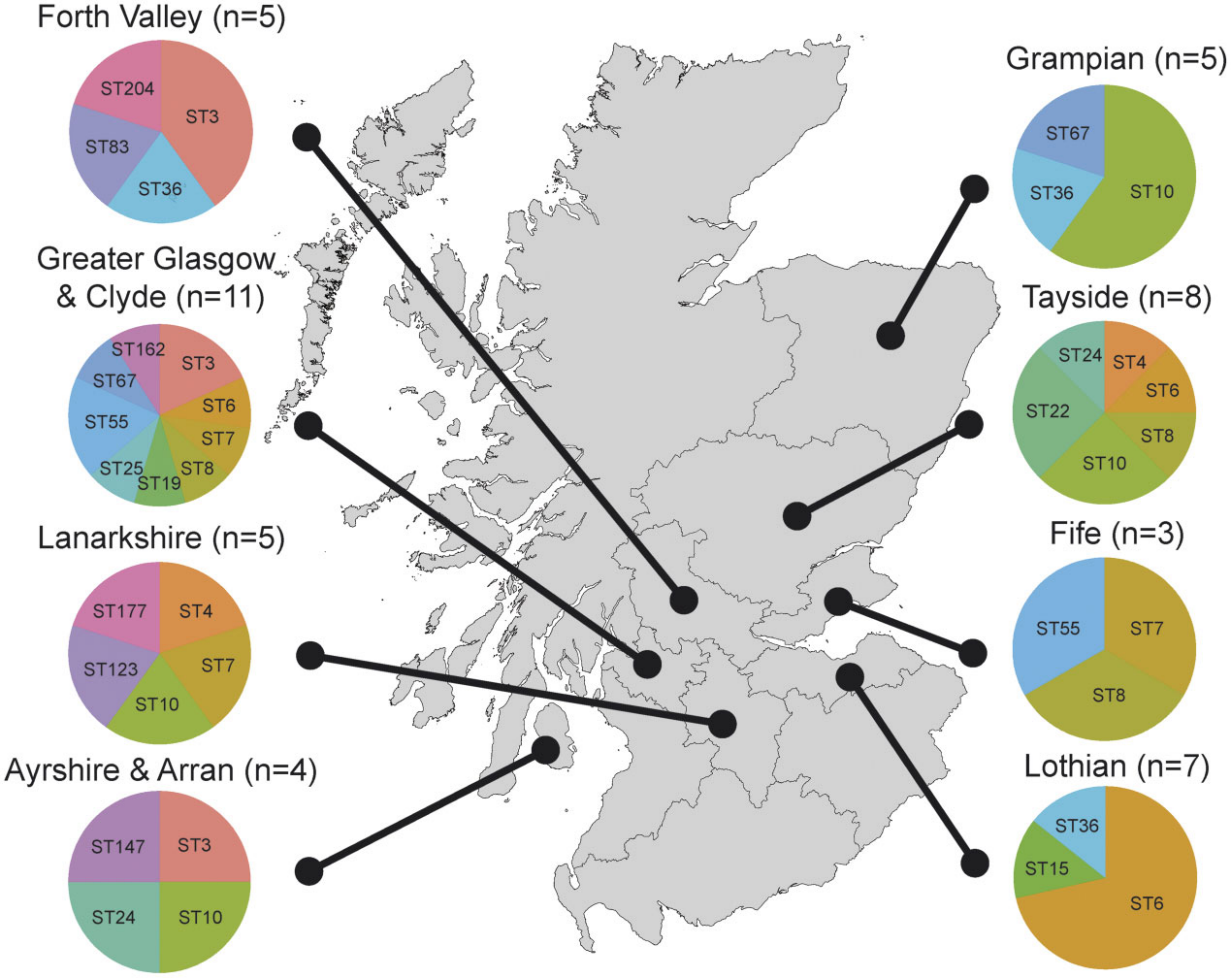
*Candida glabrata* isolates were collected across 8 health boards across Scotland in 2012, belonging to 20 separate sequence types, including the newly described ST204. Duplicate isolates stemming from the same patient at different time points have been excluded.

Clinical isolates of *C.* *glabrata* from across Scotland are highly genetically diverse. Using whole-genome sequencing, we analyzed the genomes for 68 isolates of *C. glabrata* from 47 separate patients across 8 Scottish hospitals, generating the largest panel of *C. glabrata* genome sequences to date. These 68 isolates belonged to 20 separate sequence-types (STs) of *C. glabrata*, which represent genetically related sub-populations based on alleles from 6 loci/genes. One isolate (CG57 from a single patient in Forth Valley Royal Hospital) belonged to a new ST that has not been previously identified anywhere else (ST204) (Fig. 1, Supplementary Tables 1 and 2). Variant calling using the diploid model of GATK found few examples of heterozygosity (<0.41 per kb for every isolate) suggesting all isolates were haploid (Supplementary Table 3). Our panel of *C. glabrata* isolates was supplemented with a further 83 genomes from 3 recent studies of global *C. glabrata* isolates (Biswas *et al.* 2018; Carreté *et al.* 2018; Xu *et al.* 2020), as well as sequences from the outgroup *C. bracarensis* (Correia *et al.* 2006), which has also been identified from clinical settings and is the closest known relative of *C. glabrata* (Gabaldón *et al.* 2013).

**Fig. 2.**
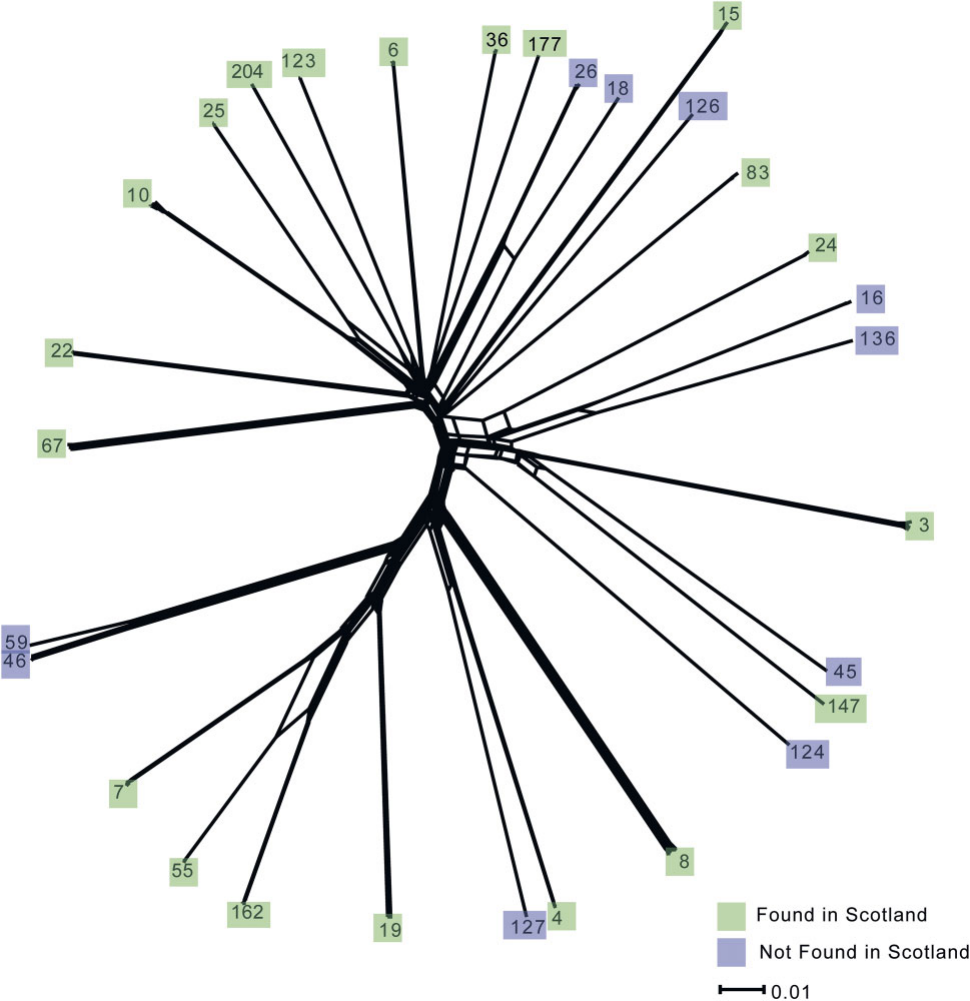
A NeighborNet network using SplitsTree, with ST labels replacing isolate names at the nodes. Green = found in Scotland, purple = not found in Scotland. The scale bar represents nucleotide substitutions per site.

**Fig. 3.**
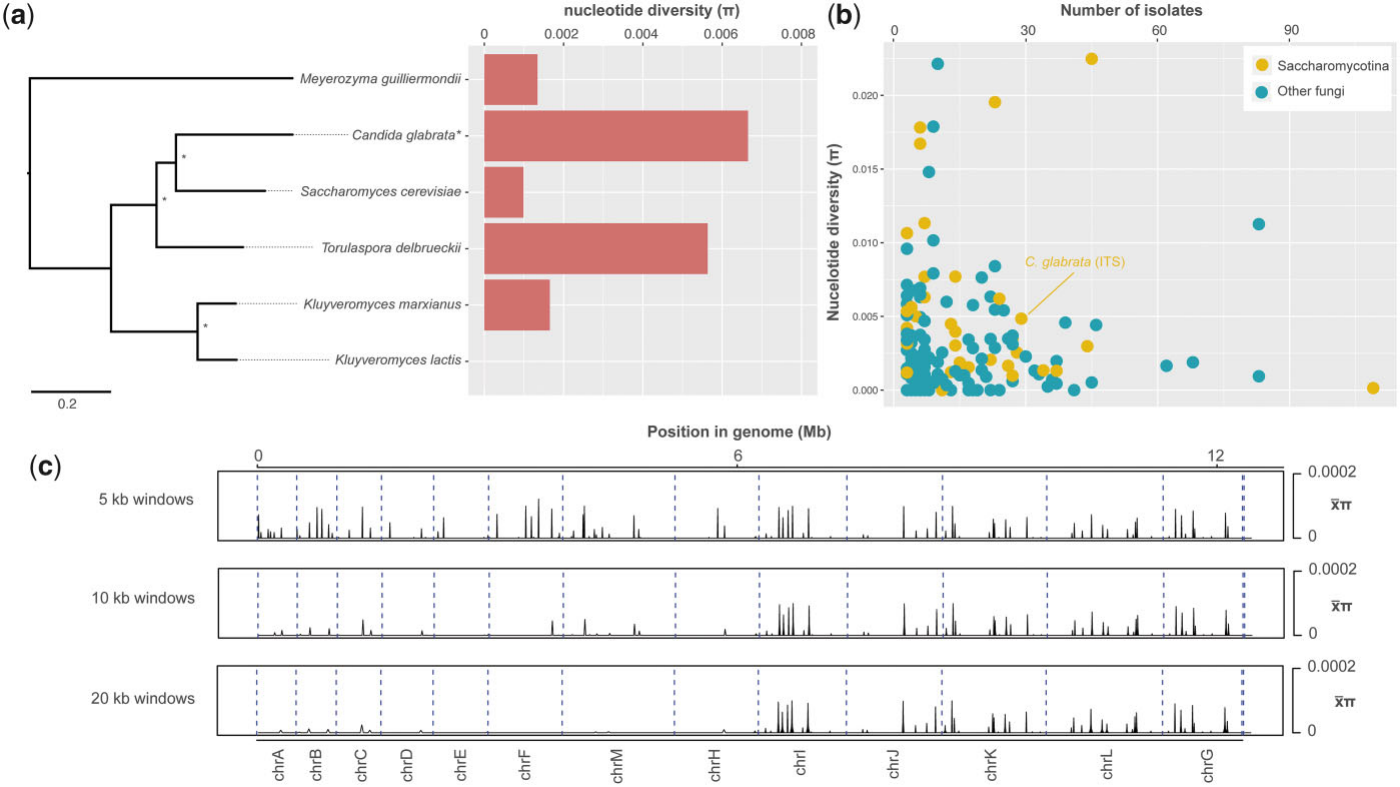
a) A RAxML phylogenetic tree with 1000 bootstrap support of all Saccharomycetaceae species that had a genome assembly in NCBI or JGI Mycocosm and nucleotide diversity (π). Note: *C. glabrata* is calculated from whole-genome sequence data presented in this study, while the other species are based on ITS sequences only (Irinyi *et al.* 2015). b) π (based on ITS sequences only) for all Saccharomycotina and non-Saccharomycotina that are listed in the ISHAM ITS reference DNA barcoding database (Irinyi *et al.* 2015). c) nonoverlapping 5, 10, and 20 kb windows of *$\bar{\text{x}}$*π (π for all sites in the genome divided by window length).

Phylogenetic analysis of our Scottish collection along with the worldwide *C. glabrata* isolates revealed high genetic diversity among the 29 separate STs represented by our combined panel (Fig. 2, Supplementary Fig. 1). Allelic diversity among *C. glabrata* isolates [mean nucleotide diversity (π) = 0.00665, σ  =  0.047  was higher than previously reported [π  =  0.00485 based on the Internal Transcribed Spacer (ITS) of 29 strains (Irinyi *et al.* 2015)]. Our WGS-based calculation of *C. glabrata* π was the highest of any species in the Saccharomycetaceae that had both an available genome assembly and a calculation of π (albeit those are based on ITS sequences and fewer strains than we had) (Irinyi *et al.* 2015) (Fig. 3a). However, *C. glabrata* genetic diversity was typical among the Saccharomycotina (mean/*$\bar{\text{x}}$* = 0.0055, median = 0.0039, standard deviaton/σ  =  0.0055) (Fig. 3b). Nucleotide diversity within the population was present across the nuclear genome (Fig. 3c), with window length having some impact on the result [smaller window lengths (5 kb) resulted in higher average π in approximately half of the genome: chromosomes A–F, M, and H]. Most of the allelic diversity across the 151 *C. glabrata* isolates came from the nuclear genome (min.  = 0.09 SNPs/kb, max.  = 6.54 SNPs/kb, *$\bar{\text{x}}$* = 5.55 SNPs/kb) compared with the mitochondrial genome (min.  = 0.05 SNPs/kb, max.  = 3.64 SNPs/kb, *$\bar{\text{x}}$* = 1.21 SNPs/kb). Indeed, a significant difference between nuclear SNPs/kb and mitochondrial SNPs/kb was found using a 2-tailed *t*-test for all 151 genomes (*P *=* *5.6147E-111).

Seven clade (C) delineations for *C. glabrata* were recently proposed (Carreté *et al.* 2018), which were equivalent to pre-existing STs including C1 (ST19), C2 (ST7), C3 (ST8), C4 (ST22), C6 (ST136), and C7 (ST3). We found that C5 was polyphyletic, encompassing isolates belonging to the genetically divergent ST6, ST10, and ST15 (Supplementary Fig. 1). Therefore, we recommend the use of ST delineations rather than those clade delineations.

Several *C. glabrata* STs had evidence of genetic recombination. Our neighbor-net network tree of all isolates suggested historic gene-flow between several STs including for example ST7, ST55, and ST162 (Fig. 2). Genomic regions with low Wright’s fixation index (*F_ST_*) values, consistent with genetic exchange, were also identified from pairwise comparisons (*n *=* *435) across 5- and 10-kb nonoverlapping windows of all STs (Supplementary Fig. 2). *F_ST_* values calculated from 5-kb windows were slightly lower than those calculated from 10-kb windows (averaging −0.046 for each ST pairwise comparison), indicating that window length impacts this measure of genetic variation. Twelve pairwise comparisons from 10-kb windows had *F_ST_ *<* *0.9 across the genome (Supplementary Fig. 3), with the lowest for ST18 and ST26 (*F_ST_ *=* *0.64). In addition, ST7, ST55, and ST162 had lower *F_ST_* values across the genome (*F_ST_ *=* *0.65–0.83) than other pairwise comparisons demonstrating incompatible phylogenetic signals between these STs (Supplementary Fig. 2).

**Fig. 4.**
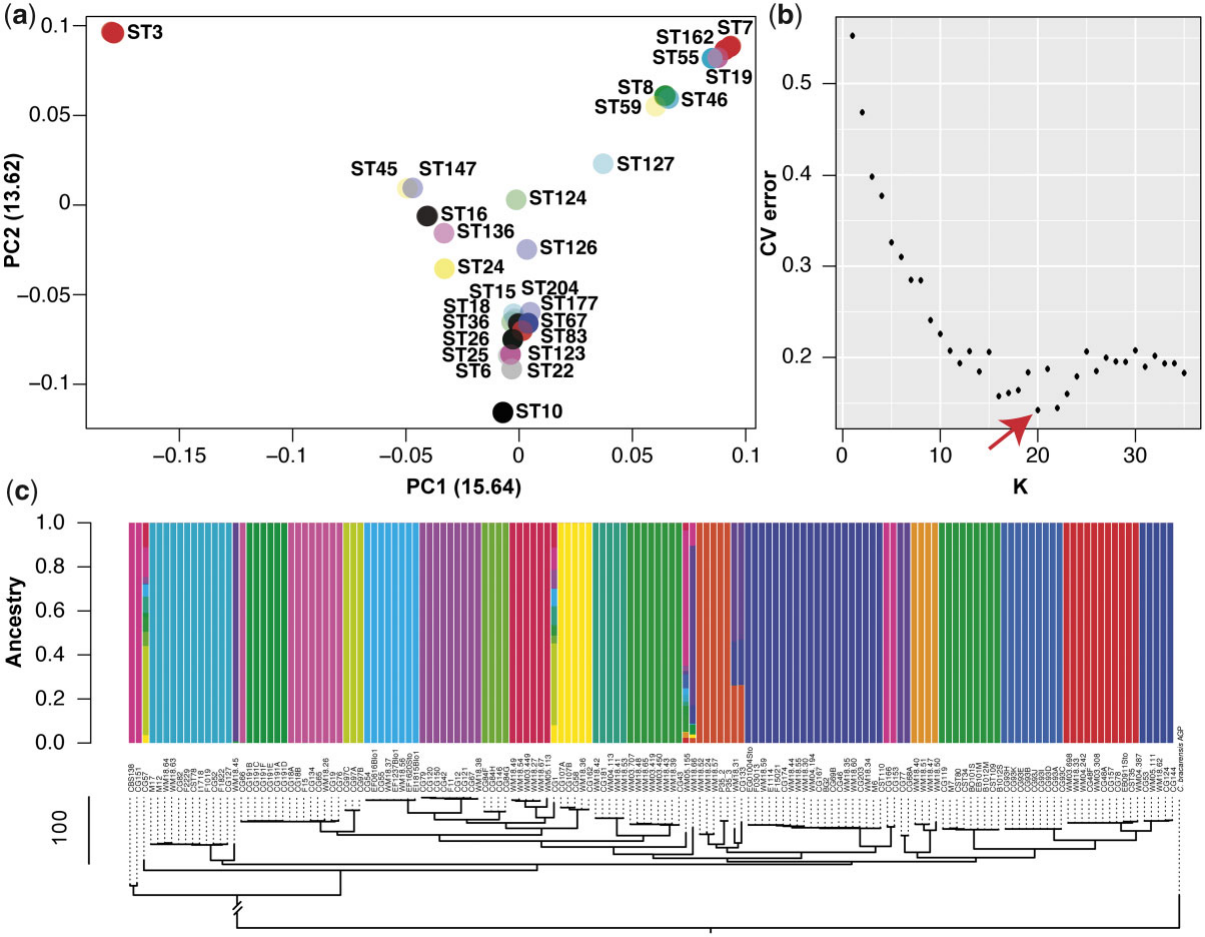
Population genetics of *C. glabrata* ST. a) PCA of whole-genome SNPs using SmartPCA revealed little evidence of sub-clustering among STs (isolates are calculated and plotted individually, but labeled by their ST alone for clarity). SmartPCA failed to calculate the eigenvalues for some isolates including those belonging to ST4. b) The CV error from running unsupervised ADMIXTURE for variant-sites across the *C. glabrata* population, testing *K*-values between 1 and 35. *K* = 20 provided the lowest CV error. c) ADMIXTURE plot for all isolates using *K* = 20, revealing several isolates with evidence of mixed ancestry. Isolates are ordered according to the neighbor-joining tree constructed with PAUP in Supplementary Fig. 1.

PCA of whole-genome SNPs revealed little evidence of clustering of several *C. glabrata* STs, which is consistent with gene flow between them (Fig. 4a). For unsupervised model-based clustering with ADMIXTURE, we first identified that *K *=* *20 had the lowest cross-validation error (Fig. 4b), and was therefore used for subsequent analysis. Two isolates were consistently (6 independent Admixture runs) found to have evidence for mixed ancestry: ST177 CG1 and our newly discovered ST204 CG57 (Fig. 4c, Supplementary Fig. 4). Other isolates were found to have evidence of mixed ancestry in the majority of runs including ST124 WM18.66, ST126 WM05.155, and ST8 M17.

Only one of the Scottish isolates (CG46) had evidence for Chromosome Copy Number Variation (CCNV)/aneuploidy, found in chromosome C (Supplementary Fig. 5). Distributions of normalized chromosome read depths of chromosome C (average depth per 10 kb window = 0.68) differ significantly from the rest of the genome of CG46 (average depth per 10 kb window = 1.05; Kolmogorov–Smirnov test: *P *=* *2.09E-25), with coverages of chromosome C significantly lower than in the rest of the genome (Wilcoxon rank-sum test: *P *=* *1.353E-25). No other CCNVs were found, despite many isolates having been treated with antifungals that have previously been correlated with CCNV (Carreté *et al.* 2019). Together, these results suggest occasional genetic recombination within the *C. glabrata* population, without an association with aneuploidy.

Mating types and mating-type switching are poorly understood in *C. glabrata*, although it is thought that Mating-type regulatory protein α2 is expressed in all MTLα strains and not in MTLa strains (Srikantha *et al.* 2003; Butler *et al.* 2004). MATα2 (CAGL0B01265g) was present in all Scottish isolates (breadth of coverage; BOC > 87%). However, MATα1 appeared to be absent or partially absent in 3/9 ST6 isolates (CG12 = 16% BOC, CG121 = 18% BOC, CG42 = 12.5% BOC), while present in the remaining 6 ST6 isolates, and all the other STs (BOC 100%). The functional relevance of MATα1 deletion or truncation is unclear but may be a hallmark of the rarer of the 2 mating types.

### Hypervariable mitochondrial genomes among sequence types

*F_ST_* analysis highlighted the mitochondrial genome of *C. glabrata* as hyper-variable (Supplementary Table 2, Supplementary Fig. 6). Forty-three genes were identified in ≥10 pairwise *F_ST_* comparisons, including all 11 mitochondrial protein-encoding genes. To explain this enrichment of low *F_ST_* mitochondrial genes, we studied the 151 genome alignments. While the nuclear genome had 97.3–99.4% BOC, the mitochondrial genome had 20.4–99.9% BOC, with 42% of isolates (*n *=* *63/151) containing >10% ambiguous mitochondrial bases (2 kb) (Supplementary Table 2) (here, we define ambiguous as sites with too few reads aligning to be called by GATK, or reads that cannot be called by GATK due to not passing variant filtration). Surprisingly, a pattern of low and/or patchy read coverage was identified in every isolate including the ST15 reference isolate CBS138 (Supplementary Fig. 6), indicating that the reference mitochondrial sequence assembly (Koszul *et al.* 2003) may have a high error rate, and given additional differences identified in nonreference isolates, that *C. glabrata* mitochondrial genomes are highly heterogenous.

**Fig. 5.**
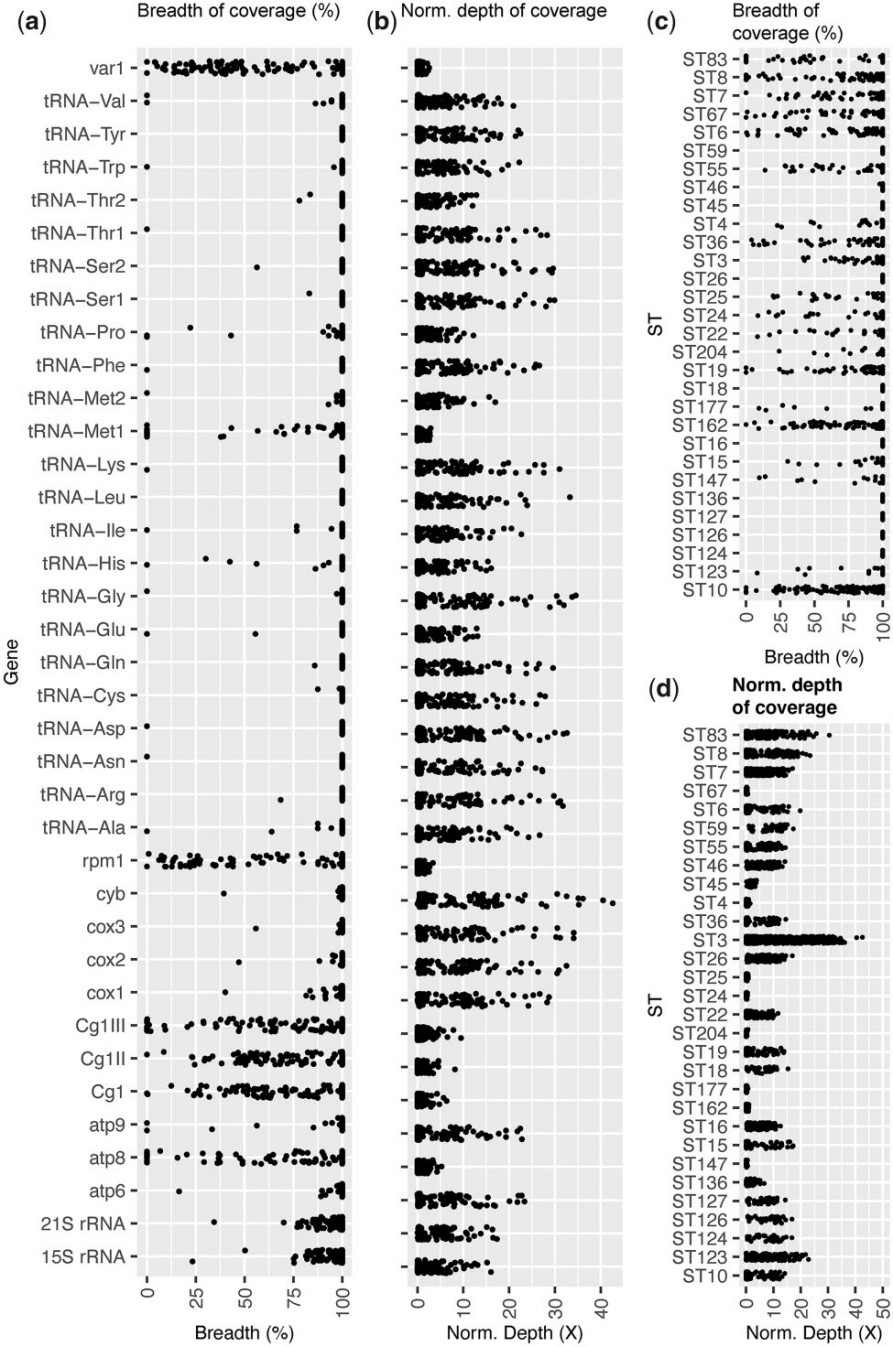
Breadth of coverage and depth of coverage across each of the 37 mitochondrial encoded genes for all 151 isolates compared in this study (Each point represents an isolate). a) Breadth of coverage as a % across each gene. b) The normalized depth of coverage for each gene (total read depth for each gene/total read depth across both nuclear and mitochondrial genomes). c) Breadth of coverage as a % across each gene, categorized by sequence types (ST)s. d) Normalized depth of coverage for each gene, categorized by ST.

The mitochondrial genome for some *C. glabrata* isolates appears reduced in size and encodes fewer protein-encoding genes (Fig. 5). As many as 22/37 (59%) mitochondrial encoded genes were entirely absent in at least 1 isolate, including Cg1, Cg1II, and Cg1III (putative endonucleases of exons and introns in the mitochondrial COX1 gene), *ATP8* and *ATP9* (subunits 8 and 9 of the enzyme complex required for ATP synthesis), *RPM1*/*RPR1* (RNA component of mitochondrial RNAse P), *VAR1* (putative mitochondrial ribosomal protein of the small subunit,) and most of the tRNA genes (15/23). Nine separate STs had absent mitochondrial genes. Normalized depth of coverage was variable across the genes, with < 1 average normalized depth across all isolates for Cg1, Cg1II, and Cg1III, *ATP8, RPM1*, *VAR1*, and tRNA-Met1. While nonuniform coverage in terms of depth and breadth was found across all mitochondrial genomes belonging to all datasets, our newly sequenced isolates have the lowest mean breadth across mitochondrial genes (*$\bar{\text{x}}$* = 92.18, σ = 20.07) compared with Biswas *et al.* (2018) (*$\bar{\text{x}}$* = 98.48, σ = 11.47) and Carreté *et al.* (2019) (*$\bar{\text{x}}$* = 97.03, σ = 14.11), suggesting there are some discrepancies between library preparation impacting mitochondrial read sequencing.

Notably, only 1/50 Biswas *et al.* (2018) isolates (WM03.450) had entirely absent mitochondrial genes compared with 8/32 Carreté *et al.* (2019) isolates and 15/68 of our newly sequence isolates. Total sequencing depth can be ruled out as the main cause for low mitochondrial coverage, given Carreté *et al.* (2019) had the highest sequencing depth (*$\bar{\text{x}}$* = 360X) and had many isolates with absent mitochondrial genes, compared with Biswas *et al.* (2018) (*$\bar{\text{x}}$* = 74X) and ours (*$\bar{\text{x}}$* = 42X).

We used assembly de novo to further explore the mitochondrial sequence for isolate WM03.450 (ST83), which had the greatest number of ambiguous bases across its mitochondrial genome (16 kb/80%). Our WM03.450 Illumina-based assembly (12.9 Mb; N.contigs = ∼3 thousand; N_50_ = 85 kb) is 562 kb longer than the CBS138 reference sequence, indicating substantial genomic differences between these isolates and STs. Aligning our assembly to the reference CBS138 mitochondrial genome using Blastn identified 10 contig matches with a combined alignment length of only 1.9 kb (mean 157 nt per contig), suggesting the low alignment is not due to conserved nucleotide sequences that have undergone large rearrangements. Aligning the assembly to the 11 mitochondrial protein sequences using Blastx identified only 6/11 genes across 6 separate contigs, 4 of which were <364 nt length, and 2 that are 10.4 and 81.5 kb. Conversely, assembly de novo and Blastx of our Illumina reads for the reference isolate CBS138 against the published CBS138 genome identified all 11 mitochondrial genes present on 4 contigs, with 18.9 kb total sequence length, of which Blastn aligned 9.3 kb to the published mitochondrial assembly. Together, these analyses suggest that whole gene deletions in the *C. glabrata* mitochondria are common.

### Signatures of selection identified among sequence types

In contrast to the *C. glabrata* mitochondrial genome, we found that gene deletions in the nuclear genome are rare. Indeed, fewer than 6 presence/absence (P/A) polymorphisms (strictly defined as zero reads aligning) were identified per isolate (∼0.1% of 5,210 protein-encoding genes) (Supplementary Table 4). Of these, 2 consecutive nuclear-encoded genes (CAGL0A02255g and CAGL0A02277g) on chromosome A were entirely absent of read coverage in 25 out of the 68 Scottish isolates (37%), which included all representatives of 11 separate STs (ST4, 7, 8, 24, 25, 55, 67, 83, 177, and our newly described 204). These STs do not cluster phylogenetically, ruling out a single evolutionary event causing this deletion. The 2 genes have identical nucleotide sequences and encode the same amino acid sequence, which is conserved across a range of other Ascomycota species, as well as having sequence similarity to the K62 Killer Preprotoxin protein encoded by the *Saccharomyces paradoxus* L-A virus M62 satellite (BLASTp *E*-value = 1e-36), suggesting a possible viral origin. CAGL0F09273g is a separate, putative adhesin-like protein (adhesin cluster V) with a “hyphally regulated cell wall protein N-terminal” PFAM that is lost in 11 isolates including all ST4 (CG68A and CG77), 4 ST7 (CG157, CG48A, CG48F, and CG78), 3 ST8 (CG127, CG52, and CG82), ST19 CG119, ST24 CG166, and ST147 CG133. Again, this gene must have been lost multiple times, given its presence in several ST7 and ST8 isolates. This gene is the last gene on chromosome F, has an π = 0.00244, which is lower than the overall average across the genome, and has previously been reported to undergo CCNV within serial clinical isolates (Carreté *et al.* 2019), suggesting it is able to undergo variation within microevolutionary timescales, which may impact the adhesive ability of these *C. glabrata* isolates.

Between 61 and 85 genes with a signature of positive selection [*dN*/*dS* = ω, and ω > 1 (Kryazhimskiy and Plotkin 2008)] were found in each ST apart from the reference ST (ST15 CG151), for which only a single gene with ω > 1 (ω = 1.0019) was identified (Supplementary Table 5). Apart from the reference ST, STs had between 4 and 14 genes with ω > 2, showing stronger signatures of diversifying or positive selection. Of the 2,083 total genes with ω > 1 across all clades, 608 were unique genes (11.6% of all genes) i.e. they had this signature in multiple clades, owing to either ancestry or selection acting on the same gene families. To explore selection, we took an unbiased approach using PFAM and GO-term enrichment comparing the numbers of each term in those 608 genes compared with the remaining nonselected genes, as well as a targeted approach for genes of interest (see *Materials and methods*: Selection and microevolutionary analysis) including adhesins, proteases, efflux pumps, FKS, and ERG pathway genes.

Genes with signatures of positive selection within the *C. glabrata* population targets diverse genes and gene functions. Our unbiased approach for enrichment of functional domains in 608 gene products with signatures of positive selection identified only 3 significantly enriched [2-tailed Fisher exact test with FDR-corrected *P*-values (q) of* *<* *0.05] PFAM domains and 16 GO terms (Table 1). The enriched PFAM domains were (1) Flocculin repeat (PF00624.20; *q *=* *7.42E-17), (2) GLEYA domain (PF10528.11; *q *=* *0.01), and (3) Armadillo repeat (PF00514.25; *q *=* *0.02). Flocculin is a sub-telomeric gene family involved in flocculation or cell aggregation in *S. cerevisiae* (Teunissen and Steensma 1995), while GLEYA domains are present in *C. glabrata EPA* proteins. Thirty Flocculin PFAM domains were assigned to only 6 genes in *C. glabrata*, 2 of which have ω > 1: CAGL0I07293g and CAGL0I00220g, and together account for 23/30 Flocculin repeat PFAMs. Enriched GO-terms covered a range of possible biological functions including ribosomal/RNA-binding and mitochondrial structural proteins.

Our targeted approach highlighted 21/129 genes of interest that have ω > 1, with at least 1 found in every ST apart from the reference ST15 and ST46 (Supplementary Table 6). Notably, none of the aspartic proteases, phospholipases, cell wall biogenesis, efflux pumps, ergosterol biosynthesis pathway genes or *FKS* genes were found to have hallmarks of positive selection, implying these are conserved within the population. Several genes with ω > 1 were found in multiple STs, including adhesive protein CAGL0J01727g (adhesin cluster VI) that is under positive selection in 7 STs (18, 26, 36, 45, 147, 177, and 204) and adhesive protein CAGL0I07293g (adhesin cluster V) under positive selection in 7 mostly distinct STs (3, 8, 25, 83, 123, 136, and 177). *C. glabrata* encodes 17 putative adhesive proteins without N-terminal signal peptides, casting doubt on their role in adhesin. One of these is a pseudogene (CAGL0E00110g) with ω > 1 in 13/29 STs. The structural cell wall protein *AWP7* belonging to the Srp1p/Tip1p family was under selection in 7 STs.

### *Candida glabrata* nosocomial in-patient microevolution targets pathogenicity factors and drug targets

**Fig. 6.**
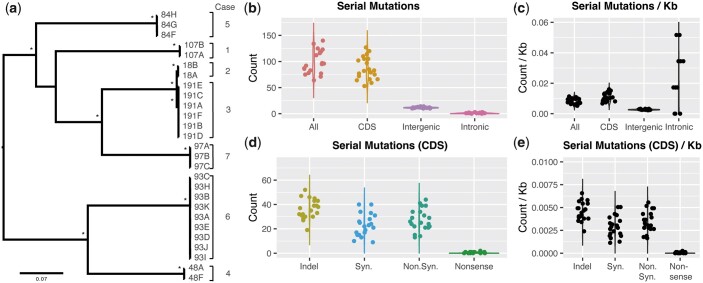
Microevolutionary changes across 7 sets of *C. glabrata* isolates. a) a RAxML phylogenetic tree of the serial isolates using the general-time-reversible model and CAT rate approximation with 100 bootstrap support. Branch lengths indicate the mean number of changes per site. b) The number of serial mutations total (All), those within protein-coding sequence (CDS), intergenic and intronic regions. c) Those same serial mutations per kb [calculated as the count of serial mutations divided by the total length of the feature (where All = whole genome) and multiplied by 1,000]. d) Serial mutations within CDS categorized by their effect on the sequencing: Insertion/Deletion (Indel), synonymous mutation (Syn.), nonsynoynmous mutation (Non.Syn.), and nonsense mutation. e) Those same serial mutations within CDS per kb.

Our Scottish *C. glabrata* panel included 7 sets of between 2 and 9 isolates from recurrent cases of candidiasis. To explore the microevolution of *C. glabrata* within a human host, and the effects of antifungal treatment (fluconazole, nystatin, and posaconazole) on fungal genetics, we documented all genetic changes between serial isolates (Table 2, Supplementary Table 7). Although exact dates of isolation were not documented, phylogenetic analysis (Supplementary Fig. 1, Fig. 6) confirmed these serial isolates were highly related, with between 64 and 140 mutations (1.13468 × 10^−5^ per base pair) identified between pairs of serial isolates (Fig. 6). While the mutation rate or generation time for *C. glabrata* is not known (Carreté *et al.* 2019), this small number of mutations likely suggests recent clonal origins appropriate for microevolutionary analysis. Serial isolates had an estimated time between isolation (based on blood culture dates) between 0 and 239 days (mean 15 days). Five serial isolates from 4 separate patients/cases showed MIC changes from the earlier sampled isolate (Table 3), including 2 increases (CG107A–B + 8 µg/ml, CG97B–C + 4 µg/ml), 1 decrease (CG84G–H −4 µg/ml,) and 1 large transient increase (CG93A–E = 4 µg/ml; CG93H, I, K > 64 µg/ml, CG93K = 4 µg/ml).

**Table 2:** Summary of microevolution across 7 sets of between 2 and 9 *C. glabrata* isolates from recurrent cases of candidiasis.

Case ID	Initial	Relapse	ST	All Mutations	Coding	Noncoding	Coding Indel	Coding Indel (frameshift)	Coding (Non.Syn.)	Coding Nonsense	Coding (Syn.)	Coding (revert to ref.)
1	CG107A	CG107B	36	97	83	14	18	9	16	0	17	32
2	CG18A	CG18B	10	140	127	13	20	7	24	0	20	63
3	CG191A	CG191B	10	64	53	11	13	4	11	0	8	21
3	CG191B	CG191C	10	78	66	12	14	9	10	0	8	34
3	CG191C	CG191D	10	83	72	11	17	12	14	0	10	31
3	CG191D	CG191E	10	96	85	11	14	4	12	0	9	50
3	CG191E	CG191F	10	87	77	10	23	16	19	0	17	18
4	CG48A	CG48F	7	92	79	13	10	3	11	0	13	45
5	CG84F	CG84G	67	76	64	12	17	5	8	0	5	34
5	CG84G	CG84H	67	71	59	12	21	5	9	0	3	26
6	CG93A	CG93B	162	125	114	11	20	4	15	0	16	63
6	CG93B	CG93C	162	119	105	14	23	10	23	0	18	41
6	CG93C	CG93D	162	124	110	14	19	7	16	0	9	66
6	CG93D	CG93E	162	112	97	15	24	10	20	1	12	40
6	CG93E	CG93H	162	119	105	14	21	5	20	0	18	46
6	CG93H	CG93I	162	116	102	14	20	6	13	0	14	55
6	CG93I	CG93J	162	96	82	14	22	13	13	0	11	36
6	CG93J	CG93K	162	135	120	15	21	11	22	1	28	48
7	CG97A	CG97B	25	79	66	13	14	3	15	0	10	27
7	CG97B	CG97C	25	86	75	11	11	4	12	0	18	34

We documented 1,995 mutations between all serial isolates, which were either in protein-coding regions (Coding) or Intergenic and intron regions (noncoding). Coding mutations were further characterized into Coding Indels, some of which caused frameshifts [Coding Indel (frameshift)], nonsynonymous mutations (Coding Non.Syn.), nonsense mutations, (Coding Nonsense), synonymous mutations (Coding Syn.), and bases that reverted back to the ST15 CBS138 reference base (either from a previous microevolutionary change or a pre-existing variant between the initial isolate and the reference ST15 CBS138).

**Table 3:** MIC values of fluconazole for each of the serial isolates.

Case ID	Strain	MIC (µg/ml)	Change
1	107a	8	
1	107b	16	8
2	18a	8	
2	18b	8	
3	191a	8	
3	191b	8	
3	191c	8	
3	191d	8	
3	191e	8	
3	191f	8	
4	48a	4	
4	48f	4	
5	84f	8	
5	84g	8	
5	84h	4	−4
6	93a	4	
6	93b	4	
6	93c	4	
6	93d	4	
6	93e	4	
6	93h	>64	>60
6	93i	>64	
6	93j	>64	
6	93k	4	−60
7	97a	4	
7	97b	4	
7	97c	8	4

Mutations identified between serial isolates were mostly in protein-coding sequence (CDS) regions (between 53 and 127 mutations per pairs of serial isolates, collectively adding up to 1,741/1,995 total mutations = 87%), despite protein-coding regions taking up only 7.9/12.3 Mb (64%) (Fig. 6, b and c). The remaining serial mutations were within intergenic regions (236 mutations; 12%) and intronic regions (18 mutations; 1%). Intronic regions had the highest count of serial mutations after accounting for the total sequence in introns (Fig. 6c), albeit with ≤3 found per pair of serial isolates. Hypergeometric tests revealed that the number of mutations in coding sequence compared with noncoding sequence was higher than expected by chance, suggesting a highly significant enrichment of mutations in protein-coding genes (*P *=* *3e-120).

To explore the 1,741 microevolutionary changes within coding regions, we categorized them into 5 groups of newly arising mutations (regardless of prior state): (1) insertions/deletions (indels) (*n *=* *362; 21%), (2) synonymous mutations (*n *=* *264; 15%), (3) nonsynonymous mutations (*n *=* *303, 17%), (4) nonsense mutations (*n *=* *2), and (5) reversion back to reference base (*n *=* *810; 47%). Of the indels, 147/362 (41%) were frameshifts that disrupted 54 genes. Nonsynonymous mutations were detected in 139 genes (Fig. 6, d and e).

Enrichment for PFAM/GO-terms of these genes with frameshift and nonsynonymous mutations [2-tailed Fisher exact test with FDR-corrected *P*-values (*q*) of < 0.05] revealed 3 enriched GO-terms and 8 enriched terms (Table 1). Both categories (frameshifts and nonsynonymous mutations) were enriched for GO: 0009986 cell surface (*q *=* *3.21E-08 and 1.09E-06, respectively), suggesting that *C. glabrata* undergoes rapid mutations in several of its cell surface proteins during prolonged/serial blood stream infections. Enriched PFAM terms included the “RNA polymerase *RPB1* C-terminal repeat” for genes with frameshift indels (*q *=* *1.25E-36), GLEYA domains for genes with either frameshift (*q *=* *3.15E-12) or nonsynonymous mutations (*q *=* *3.58E-08). Several repeat-associated PFAMs and the “Hyphally regulated cell wall protein N-terminal” domain were enriched for nonsynonymous mutations (*q *=* *5.87E-04).

Several genes of interest (see *Materials and methods*: Selection and microevolutionary analysis) had microevolutionary changes (*n *=* *29/129) (Supplementary Table 8). Notably, 1 of the 2 newly acquired nonsense mutations was identified in *FKS2* (Case 6 J–K), coinciding with a substantial drop in fluconazole MIC (Table 3). The other was in the uncharacterized CAGL0K04631g at an earlier time point in the same patient (Case 6 D–E).

Twenty adhesins including *EPA* genes were mutated between serial isolates, including in all 7 sets/cases of isolates and at every time point. For example, *EPA3* had 5 indels in Case 1 (A–B), a synonymous mutation in Case 2 [A–B; nucleotide position (pos.) 2304], Case 3 (D–E; pos. 1539), Case 4 (A–F; pos. 1119), Case 5 (F–G; pos. 2259), a nonsynonymous mutation (pos. 2224) and large (30 nt) insertion in Case 5 (G–H), 2 large deletions (42 and 16 nt), and 2 synonymous and 1 nonsynonymous mutations in Case 6 (A–B; pos. 1002, 2319 and 2276, respectively).

The longer 42 nt deletion from Case 6 (A–B) reverts back to reference in Case 6 (B–C), suggesting either (1) a nondescendent isolate (intra-host variation), (2) a false-negative reference in 6C or (3) a false-positive deletion in 6A. The same 42 nt deletion, along with a new insertion at the site of the previous synonymous mutation appears in Case 6 (C–D), thereby suggesting the variant is real and option c less likely. That 42 nt deletion reverts back to reference in Case 6 (D–E), and appears again in Case 6 (E–H). By Case 6 (H–I), the gene has a new synonymous mutation, and in Case 6 (I–J) it has accumulated a new 15 nt deletion. *EPA3* is therefore a hot-spot of variation. Another EPA gene that accumulated a large number of mutations was *AWP12*, which accumulated 5 nonsynonymous mutations and 1 synonymous mutation (Case 6H–I).

Other genes that had accumulated mutations between serial isolates included those encoding an aspartic protease *YPS5*, several structural wall proteins belonging to the Srp1/Tip family, regulatory protein *PDR1*, the ergosterol synthesis gene *ERG4* (a nonsynonymous mutation in Case 3A–B), and both *FKS1* and *FKS2*. Therefore, *C. glabrata* genes that are antifungal targets and gene families involved in drug-resistance and pathogenicity can therefore undergo rapid mutation within a human host.

## Discussion

In this study, we sequenced and analyzed the largest panel of *C. glabrata* genomes to date. These isolates were collected from blood-stream infections of patients at several Scottish hospitals in 2012. Our 68 genomes were analyzed alongside 83 further publicly available and globally isolated genomes (Biswas *et al.* 2018; Carreté *et al.* 2019; Xu *et al.* 2020), revealing greater genetic diversity than previously recognized, including a nucleotide diversity of 0.00665, which is much higher than has been calculated for the distantly related *C. albicans* at 0.00298 (Irinyi *et al.* 2015). Surprisingly, we found that only one of our Scottish isolates had evidence of aneuploidy, despite many having been treated with antifungals, which has previously been correlated (Carreté *et al.* 2019). Chromosome C in CG46 had lower depth of coverage compared with the rest of the genome, perhaps due to chromosome loss in a subset of cells. The patient that CG46 was isolated from was initially treated with Fluconazole. Following resistance to Fluconazole being detected, the patient was subsequently treated with Caspofungin, suggesting a potential link between those antifungal treatments and the observed aneuploidy.

We found that the mitochondrial genome of *C. glabrata* was hyper-diverse compared with its nuclear genome for many isolates, including several long deletions spanning one or more genes, with the potential to impact many important biological functions including drug resistance and persistence (Garcia-Rubio *et al.* 2021). High levels of variation in mitochondrial genomes within the major fungal phyla have previously been noted in terms of gene order, genome size, composition of intergenic regions, presence of repeats, introns, associated ORFs, and evidence for mitochondrial recombination in all fungal phyla (Aguileta *et al.* 2014). This variation is lacking in Metazoa (Aguileta *et al.* 2014). Our results suggest some of these types of mitochondrial genetic diversity are likely present within the *C. glabrata* population.

Isolates in this study belonged to 29 separate STs of *C. glabrata*, each of which was separated by large number of variants. However, as many as 193 MLST STs have been documented (Jolley *et al.* 2018). Therefore, it is likely that the true genetic diversity of *C. glabrata* is much higher than we have been able to calculate with whole-genome sequences (albeit the largest panel studied to date). Indeed, several further STs may yield further evidence of recombination or lack of, and may ultimately require a new effort to group STs into lineages (also dependent on the frequency of recombination that erode these divisions). The genetic diversity of *C. glabrata* in hospitals around Scotland is extremely high, with representatives from 20 STs. Such high genetic diversity (and many of the same STs) have also been found from genome sequencing and phylogenetic analysis of isolates collected in other countries such as Australia (Biswas *et al.* 2018), suggesting they must have been transported across or between continents, perhaps by anthropogenic or even natural means [for example its association with birds (Cafarchia *et al.* 2008; Al-Yasiri *et al.* 2016) and food (de Melo Pereira *et al.* 2014)]. Greater sampling and genotyping of clinical and environmental isolates will be required for understanding ancestry or endemicity.

*Candida* *glabrata* has long been regarded as a haploid asexual yeast, although evidence has recently emerged of a cryptic sexual cycle (Wong *et al.* 2003; Dodgson *et al.* 2005; Gabaldón and Fairhead 2019). Our genome sequencing and population genetics supports this work, revealing compelling evidence that at least 12 STs stem from recombination between other STs. However, further work remains to document and describe individual recombinant isolates. Providing genetic recombination between isolates is naturally occurring, the mechanisms of genetic exchange are also unknown, although likely relate to the conserved mating-type locus, which play a central role in the sexual cycle of diverse fungi (Fraser and Heitman 2003). Here, we show that the MATα1 gene was absent or partially absent in 3 isolates belonging to ST6, which could potentially impact or even be a hallmark of a rarer second mating type of *C. glabrata*. Together, genetic recombination among *C. glabrata* isolates appears much more common than previously recognized, and likely contributes to increased genetic diversity.

The nuclear genome for isolates belonging to every ST (apart from the reference ST15 that was included as a control) included evidence of positive or diversifying selection. Signatures of positive selection were found enriched in genes with diverse functions, including several with repeat domains, as well as EPA genes with GLEYA domains. EPA genes are a large sub-telomeric family of virulence-related surface glycoprotein-encoding genes encoded by several other pathogens including *Plasmodium*, *Trypanosoma*, and *Pneumocystis* (De Las Peñas *et al.* 2003). Such gene differences between STs of *C. glabrata* may result in clinically relevant phenotypic differences.

In host microevolutionary changes between serial isolates were enriched within coding-sequences, which is a surprise, given the expectation for intergenic regions to be more permissive to mutations due to relaxed selection within intergenic regions and purifying selection within coding sequence. The reason for this abundance of serial mutations in coding sequence is unclear, although it could potentially be technical (e.g. false-negative variants within repetitive sequences) or biological (e.g. drug exposure and host immune pressure). Alternatively, enriched mutations in genes could potentially be driven by processes such as DNA polymerase induced mutations, or differences in chromatin states (e.g. heterochromatin could lead to increased exposure to DNA damaging agents resulting in higher mutation rates, or conversely, greater surveillance and correction of mutations in euchromatin regions by cellular DNA repair enzymes, Makova and Hardison 2015).

Selection may explain why we identified similar numbers of nonsynonymous mutations to synonymous mutations, given random mutations are expected to be nonsynonymous in ∼2/3 nucleotides of each codon. Furthermore, accumulations of deleterious mutations could be occurring in the serial isolates due to small population sizes, although population size estimates could not be calculated accurately from the metadata.

Genes with GLEYA domains including *EPA* genes were significantly enriched for frameshift and nonsynonymous mutations in the coding sequence between serial isolates. Combined with our finding of positive selection in EPA genes across STs, suggests that EPA genes are undergoing variation at both longer time frames and microevolutionary time-scales.

Genes encoding several important drug targets also underwent mutations between serial isolates, including a nonsynonymous mutation in the ergosterol biosynthesis pathway gene *ERG4*, and a nonsense mutation in the 1,3-β-glucan synthase component *FKS2* [mutations in these genes can confer resistance to azoles (Feng *et al.* 2017) and echinocandins (Katiyar *et al.* 2012, p. 1), respectively]. Indeed, the nonsense mutation in *FKS2* coincided with a marked drop in fluconazole MIC for isolate CG93K, suggesting a possible link.

Our study highlights the need for further sampling and genomic analysis of *C. glabrata* in order to better inform the population structure and mechanisms underlying its increasing emergence, pathogenicity and multi-drug resistance. While we have largely focused on differences among the conserved regions of the *C. glabrata* ST15 CBS138 genome using an alignment-based strategy, our discoveries of a hyper-diverse mitochondrial sequence highlight the value for future long-read sequencing and assemblies to characterize the pan-genomes of *C. glabrata* and structural genomic diversity that exists among and perhaps within STs, and to explore the mechanisms driving those changes. Furthermore, given the genetic diversity between STs that we document, it would likely be valuable to sequence and assemble additional high-quality reference sequences for the purposes of increasing variant-calling accuracy and quantifying gene content between different STs. Given the low and patchy alignment depth across the ST15 CBS138 mitochondrial sequence for that same isolate, a review and update for the published CBS138 mitochondrial genome is likely required as well. Indeed, high (∼0.5–1%) frequencies of structural variation in the nuclear genomes of *C. glabrata* isolates was recently found using de novo assemblies from long single-molecule real-time reads (Xu *et al.* 2021).

The rapidity that *C. glabrata* can mutate important genes and gene families, both *via* microevolution and putative recombination highlights an obstacle for future drug-development, given that individual gene targets are able to mutate within short time spans, and substantial diversity already present between STs. In addition, the epidemiology of *C. glabrata* is poorly understood. Future sampling and genomic comparison studies are necessary to identify the routes and mechanisms of its spread and evolution.

## Data availability

Raw sequences for all haploid isolates of *C. glabrata* from this study have been deposited in the NCBI Sequence Read Archive (SRA) under BioProject PRJNA669061.

Supplemental material is available at *GENETICS* online.

## Supplementary Material

iyac031_Supplementary_Figure_S1

iyac031_Supplementary_Figure_S2

iyac031_Supplementary_Figure_S3

iyac031_Supplementary_Figure_S4

iyac031_Supplementary_Figure_S5

iyac031_Supplemental_Material_Legends

iyac031_Supplementary_Table_S1

iyac031_Supplementary_Table_S2

iyac031_Supplementary_Table_S3

iyac031_Supplementary_Table_S4

iyac031_Supplementary_Table_S5

iyac031_Supplementary_Table_S6

iyac031_Supplementary_Table_S7

iyac031_Supplementary_Table_S8

## References

[iyac031-B1] Abraham G , Inouye M. Fast principal component analysis of large-scale genome-wide data. PLoS One. 2014;9(4):e93766.doi:10.1371/journal.pone.0093766. 24718290 PMC3981753

[iyac031-B2] Agapow P-M , Burt A. Indices of multilocus linkage disequilibrium. Mol Ecol Notes. 2001;1(1–2):101–102. doi:10.1046/j.1471–8278.2000.00014.x.

[iyac031-B3] Aguileta G , de Vienne DM, Ross ON, Hood ME, Giraud T, Petit E, Gabaldón T. High variability of mitochondrial gene order among fungi. Genome Biol Evol. 2014;6(2):451–465. doi:10.1093/gbe/evu028. 24504088 10.1093/gbe/evu028 PMC3942027

[iyac031-B4] Alexander BD ; Clinical and Laboratory Standards Institute. *Reference Method for Broth dilution Antifungal Susceptibility Testing of Yeasts*. 4th ed. https://clsi.org/standards/products/microbiology/documents/m27/. ISBN Number: 1-56238-827-4. 2017.

[iyac031-B5] Alexander DH , Novembre J, Lange K. Fast model-based estimation of ancestry in unrelated individuals. Genome Res. 2009;19(9):1655–1664. doi:10.1101/gr.094052.109. 19648217 PMC2752134

[iyac031-B6] Al-Yasiri MH , Normand A-C, L'Ollivier C, Lachaud L, Bourgeois N, Rebaudet S, Piarroux R, Mauffrey J-F, Ranque S. Opportunistic fungal pathogen *Candida glabrata* circulates between humans and yellow-legged gulls. Sci Rep. 2016;6:36157.doi:10.1038/srep36157. 27782182 PMC5080578

[iyac031-B7] Arora P , Singh P, Wang Y, Yadav A, Pawar K, Singh A, Padmavati G, Xu J, Chowdhary A. Environmental isolation of *Candida auris* from the coastal wetlands of Andaman Islands, India. mBio. 2021;12(2):e03181-20. https://doi.org/10.1128/mBio.03181-20. 33727354 10.1128/mBio.03181-20 PMC8092279

[iyac031-B8] Bankevich A , Nurk S, Antipov D, Gurevich AA, Dvorkin M, Kulikov AS, Lesin VM, Nikolenko SI, Pham S, Prjibelski AD, et al SPAdes: a new genome assembly algorithm and its applications to single-cell sequencing. J Comput Biol. 2012;19(5):455–477. doi:10.1089/cmb.2012.0021. 22506599 PMC3342519

[iyac031-B9] Barber AE , Weber M, Kaerger K, Linde J, Gölz H, Duerschmied D, Markert A, Guthke R, Walther G, Kurzai O, et al Comparative genomics of serial *Candida glabrata* isolates and the rapid acquisition of echinocandin resistance during therapy. Antimicrob Agents Chemother. 2019;63(2):e01628-18. doi:10.1128/AAC.01628-18. 30478162 10.1128/AAC.01628-18 PMC6355595

[iyac031-B10] Beck-Sagué C , Jarvis WR, Secular trends in the epidemiology of nosocomial fungal infections in the United States, 1980–1990. National Nosocomial Infections Surveillance System. J Infect Dis. 1993;167(5):1247–1251. doi:10.1093/infdis/167.5.1247. 8486965

[iyac031-B11] Bink A , Vandenbosch D, Coenye T, Nelis H, Cammue BPA, Thevissen K. Superoxide dismutases are involved in *Candida albicans* biofilm persistence against Miconazole▿. Antimicrob Agents Chemother. 2011;55(9):4033–4037. doi:10.1128/AAC.00280-11. 21746956 PMC3165342

[iyac031-B12] Biswas C , Marcelino VR, Van Hal S, Halliday C, Martinez E, Wang Q, Kidd S, Kennedy K, Marriott D, Morrissey CO, et al Whole genome sequencing of Australian *Candida glabrata* isolates reveals genetic diversity and novel sequence types. Front Microbiol. 2018;9:2946. doi:10.3389/fmicb.2018.02946. 30559734 PMC6287553

[iyac031-B13] Brunke S , Hube B. Two unlike cousins: *Candida albicans* and *C. glabrata* infection strategies. Cell Microbiol. 2013;15(5):701–708. doi:10.1111/cmi.12091. 23253282 PMC3654559

[iyac031-B14] Butler G , Kenny C, Fagan A, Kurischko C, Gaillardin C, Wolfe KH. Evolution of the MAT locus and its Ho endonuclease in yeast species. Proc Natl Acad Sci USA. 2004;101(6):1632–1637. 14745027 10.1073/pnas.0304170101 PMC341799

[iyac031-B15] Cafarchia C , Romito D, Coccioli C, Camarda A, Otranto D. Phospholipase activity of yeasts from wild birds and possible implications for human disease. Med Mycol. 2008;46(5):429–434. doi:10.1080/13693780701885636. 18608940

[iyac031-B16] Carreté L , Ksiezopolska E, Pegueroles C, Gómez-Molero E, Saus E, Iraola-Guzmán S, Loska D, Bader O, Fairhead C, Gabaldón T, et al Patterns of genomic variation in the opportunistic pathogen *Candida glabrata* suggest the existence of mating and a secondary association with humans. Curr Biol. 2018;28(1):15–27.e7. doi:10.1016/j.cub.2017.11.027. 29249661 PMC5772174

[iyac031-B17] Carreté L , Ksiezopolska E, Gómez-Molero E, Angoulvant A, Bader O, Fairhead C, Gabaldón T. Genome comparisons of *Candida glabrata* serial clinical isolates reveal patterns of genetic variation in infecting clonal populations. Front Microbiol. 2019;10:112.doi:3389/fmicb.2019.00112. 30809200 10.3389/fmicb.2019.00112 PMC6379656

[iyac031-B18] Conesa A , Götz S, García-Gómez JM, Terol J, Talón M, Robles M. Blast2GO: a universal tool for annotation, visualization and analysis in functional genomics research. Bioinformatics. 2005;21(18):3674–3676. doi:10.1093/bioinformatics/bti610. 16081474

[iyac031-B19] Cormack BP , Ghori N, Falkow S. An adhesin of the yeast pathogen *Candida glabrata* mediating adherence to human epithelial cells. Science. 1999;285(5427):578–582. doi:10.1126/science.285.5427.578. 10417386 10.1126/science.285.5427.578

[iyac031-B20] Correia A , Sampaio P, James S, Pais C. *Candida bracarensis* sp. nov., a novel anamorphic yeast species phenotypically similar to *Candida glabrata*. Int J Syst Evol Microbiol. 2006;56(Pt 1):313–317. doi:10.1099/ijs.0.64076-0. 16403904

[iyac031-B21] Cowen LE , Anderson JB, Kohn LM. Evolution of drug resistance in *Candida albicans*. Annu Rev Microbiol. 2002;56:139–165. doi:10.1146/annurev.micro.56.012302.160907. 12142485

[iyac031-B22] Danecek P , Auton A, Abecasis G, Albers CA, Banks E, DePristo MA, Handsaker RE, Lunter G, Marth GT, Sherry ST, et al; 1000 Genomes Project Analysis Group. The variant call format and VCFtools. Bioinformatics. 2011;27(15):2156–2158. doi:10.1093/bioinformatics/btr330. 21653522 PMC3137218

[iyac031-B23] Daniel H-M , Lachance M-A, Kurtzman CP. On the reclassification of species assigned to *Candida* and other anamorphic ascomycetous yeast genera based on phylogenetic circumscription. Antonie Van Leeuwenhoek. 2014;106(1):67–84. doi:10.1007/s10482-014-0170-z. 24748333 10.1007/s10482-014-0170-z

[iyac031-B24] De Las Peñas A , Pan S-J, Castaño I, Alder J, Cregg R, Cormack BP. Virulence-related surface glycoproteins in the yeast pathogen *Candida glabrata* are encoded in subtelomeric clusters and subject to RAP1- and SIR-dependent transcriptional silencing. Genes Dev. 2003;17(18):2245–2258. doi:10.1101/gad.1121003. 12952896 PMC196462

[iyac031-B25] de Melo Pereira GV , Soccol VT, Pandey A, Medeiros ABP, Andrade Lara JMR, Gollo AL, Soccol CR. Isolation, selection and evaluation of yeasts for use in fermentation of coffee beans by the wet process. Int J Food Microbiol. 2014;188:60–66. doi:10.1016/j.ijfoodmicro.2014.07.008. 25087206

[iyac031-B26] Dodgson AR , Pujol C, Pfaller MA, Denning DW, Soll DR. Evidence for recombination in *Candida glabrata*. Fungal Genet Biol. 2005;42(3):233–243. doi:10.1016/j.fgb.2004.11.010. 15707844

[iyac031-B27] Drenth A , McTaggart AR, Wingfield BD. Fungal clones win the battle, but recombination wins the war. IMA Fungus. 2019;10(1):18.doi:10.1186/s43008-019-0020-8. 32647622 PMC7325676

[iyac031-B28] Feng W , Yang J, Xi Z, Qiao Z, Lv Y, Wang Y, Ma Y, Wang Y, Cen W. Mutations and/or overexpressions of ERG4 and ERG11 genes in clinical azoles-resistant isolates of *Candida albicans*. Microb Drug Resist. 2017;23(5):563–570. doi:10.1089/mdr.2016.0095. 27976986

[iyac031-B29] Fraser JA , Heitman J. Fungal mating-type loci. Curr Biol. 2003;13(20):R792–R795. doi:10.1016/j.cub.2003.09.046. 14561417

[iyac031-B30] Gabaldón T , Martin T, Marcet-Houben M, Durrens P, Bolotin-Fukuhara M, Lespinet O, Arnaise S, Boisnard S, Aguileta G, Atanasova R, et al Comparative genomics of emerging pathogens in the *Candida glabrata* clade. BMC Genomics. 2013;14(1):623. doi:10.1186/1471-2164-14-623. 24034898 PMC3847288

[iyac031-B31] Gabaldón T , Fairhead C. Genomes shed light on the secret life of *Candida glabrata*: not so asexual, not so commensal. Curr Genet. 2019;65(1):93–98. doi:10.1007/s00294-018-0867-z. 30027485 PMC6342864

[iyac031-B32] Gabaldón T , Gómez-Molero E, Bader O. Molecular typing of *Candida glabrata*. Mycopathologia. 2020;185(5):755–764. doi:10.1007/s11046-019-00388-x. 31617105 10.1007/s11046-019-00388-x

[iyac031-B33] Garcia-Rubio R , Jimenez-Ortigosa C, DeGregorio L, Quinteros C, Shor E, Perlin DS. Multifactorial role of mitochondria in echinocandin tolerance revealed by transcriptome analysis of drug-tolerant cells. mBio. 2021;12(4):e01959-21. doi:10.1128/mBio.01959-21. 34372698 PMC8406274

[iyac031-B34] Hirano R , Sakamoto Y, Kudo K, Ohnishi M. Retrospective analysis of mortality and *Candida* isolates of 75 patients with candidemia: a single hospital experience. Infect Drug Resist. 2015;8:199–205. doi:10.2147/IDR.S80677. 26185460 PMC4501221

[iyac031-B35] Hudson RR , Kaplan NL. Statistical properties of the number of recombination events in the history of a sample of DNA sequences. Genetics. 1985;111(1):147–164. https://doi:10.1093/genetics/111.1.147. 4029609 10.1093/genetics/111.1.147 PMC1202594

[iyac031-B36] Huson DH. SplitsTree: analyzing and visualizing evolutionary data. Bioinformatics. 1998;14(1):68–73. 9520503 10.1093/bioinformatics/14.1.68

[iyac031-B37] Ibrahim AS , Mirbod F, Filler SG, Banno Y, Cole GT, Kitajima Y, Edwards JE, Nozawa Y, Ghannoum MA. Evidence implicating phospholipase as a virulence factor of *Candida albicans*. Infect Immun. 1995;63(5):1993–1998. doi:10.1128/iai.63.5.1993-1998.1995. 7729913 10.1128/iai.63.5.1993-1998.1995 PMC173255

[iyac031-B38] Irinyi L , Serena C, Garcia-Hermoso D, Arabatzis M, Desnos-Ollivier M, Vu D, Cardinali G, Arthur I, Normand A-C, Giraldo A, et al International Society of Human and Animal Mycology (ISHAM)-ITS reference DNA barcoding database—the quality controlled standard tool for routine identification of human and animal pathogenic fungi. Med Mycol. 2015;53(4):313–337. doi:10.1093/mmy/myv008. 25802363 10.1093/mmy/myv008

[iyac031-B39] Jolley KA , Bray JE, Maiden MCJ. Open-access bacterial population genomics: BIGSdb software, the PubMLST.org website and their applications. Wellcome Open Res. 2018;3:124.doi:10.12688/wellcomeopenres.14826.1. 30345391 PMC6192448

[iyac031-B40] Katiyar SK , Alastruey-Izquierdo A, Healey KR, Johnson ME, Perlin DS, Edlind TD. Fks1 and Fks2 are functionally redundant but differentially regulated in *Candida glabrata*: implications for echinocandin resistance. Antimicrob Agents Chemother. 2012;56(12):6304–6309. doi:10.1128/AAC.00813-12. 23027185 PMC3497156

[iyac031-B41] Koszul R , Malpertuy A, Frangeul L, Bouchier C, Wincker P, Thierry A, Duthoy S, Ferris S, Hennequin C, Dujon B, et al The complete mitochondrial genome sequence of the pathogenic yeast *Candida (Torulopsis) glabrata*. FEBS Lett. 2003;534(1–3):39–48. doi:10.1016/s0014-5793(02)03749-3. 12527359

[iyac031-B42] Kryazhimskiy S , Plotkin JB. The population genetics of dN/dS. PLoS Genet. 2008;4(12):e1000304.doi:10.1371/journal.pgen.1000304. 19081788 PMC2596312

[iyac031-B43] Lamoth F , Lockhart SR, Berkow EL, Calandra T. Changes in the epidemiological landscape of invasive candidiasis. J Antimicrob Chemother. 2018;73(suppl. 1):i4–i13. doi:10.1093/jac/dkx444. 29304207 PMC11931512

[iyac031-B44] Li H , Handsaker B, Wysoker A, Fennell T, Ruan J, Homer N, Marth G, Abecasis G, Durbin R, 1000 Genome Project Data Processing Subgroup. The Sequence Alignment/Map format and SAMtools. Bioinformatics. 2009;25(16):2078–2079. doi:10.1093/bioinformatics/btp352. 19505943 PMC2723002

[iyac031-B45] Li H. Aligning sequence reads, clone sequences and assembly contigs with BWA-MEM. arXiv. 2013; 1303.3997 [q-bio] [Preprint]. http://arxiv.org/abs/1303.3997 (accessed 2018 May 29).

[iyac031-B46] Makova KD , Hardison RC. The effects of chromatin organization on variation in mutation rates in the genome. Nat Rev Genet. 2015;16(4):213–223. doi:10.1038/nrg3890. 25732611 PMC4500049

[iyac031-B47] Martchenko M , Alarco A-M, Harcus D, Whiteway M. Superoxide dismutases in *Candida albicans*: transcriptional regulation and functional characterization of the hyphal-induced SOD5 gene. Mol Biol Cell. 2004;15(2):456–467. doi:10.1091/mbc.E03-03-0179. 14617819 PMC329211

[iyac031-B48] McKenna A , Hanna M, Banks E, Sivachenko A, Cibulskis K, Kernytsky A, Garimella K, Altshuler D, Gabriel S, Daly M, et al The Genome Analysis Toolkit: a MapReduce framework for analyzing next-generation DNA sequencing data. Genome Res. 2010;20(9):1297–1303. doi:10.1101/gr.107524.110. 20644199 PMC2928508

[iyac031-B49] Mullick A , Leon Z, Min-Oo G, Berghout J, Lo R, Daniels E, Gros P. Cardiac failure in C5-deficient A/J mice after *Candida albicans* infection. Infect Immun. 2006;74(8):4439–4451. doi:10.1128/IAI.00159-06. 16861630 10.1128/IAI.00159-06 PMC1539620

[iyac031-B50] Odds FC , Hanson MF, Davidson AD, Jacobsen MD, Wright P, Whyte JA, Gow NAR, Jones BL. One year prospective survey of *Candida* bloodstream infections in Scotland. J Med Microbiol. 2007;56(Pt 8):1066–1075. doi:10.1099/jmm.0.47239-0. 17644714 PMC2884937

[iyac031-B51] Perlroth J , Choi B, Spellberg B. Nosocomial fungal infections: epidemiology, diagnosis, and treatment. Med Mycol. 2007;45(4):321–346. doi:10.1080/13693780701218689. 17510856

[iyac031-B52] Pfaller MA , Castanheira M, Lockhart SR, Ahlquist AM, Messer SA, Jones RN. Frequency of decreased susceptibility and resistance to echinocandins among fluconazole-resistant bloodstream isolates of *Candida glabrata*. J Clin Microbiol. 2012;50(4):1199–1203. doi:10.1128/JCM.06112-11. 22278842 PMC3318516

[iyac031-B53] Purcell S , Neale B, Todd-Brown K, Thomas L, Ferreira MAR, Bender D, Maller J, Sklar P, de Bakker PIW, Daly MJ, et al PLINK: a tool set for whole-genome association and population-based linkage analyses. Am J Hum Genet. 2007;81(3):559–575. doi:10.1086/519795. 17701901 PMC1950838

[iyac031-B54] Rajendran R , Sherry L, Deshpande A, Johnson EM, Hanson MF, Williams C, Munro CA, Jones BL, Ramage G. A prospective surveillance study of candidaemia: epidemiology, risk factors, antifungal treatment and outcome in hospitalized patients. Front Microbiol. 2016a;7:915.doi:10.3389/fmicb.2016.00915. 27379047 PMC4910670

[iyac031-B55] Rajendran R , Sherry L, Nile CJ, Sherriff A, Johnson EM, Hanson MF, Williams C, Munro CA, Jones BJ, Ramage G, et al Biofilm formation is a risk factor for mortality in patients with *Candida albicans* bloodstream infection-Scotland, 2012–2013. Clin Microbiol Infect. 2016b;22(1):87–93. doi:10.1016/j.cmi.2015.09.018. 26432192 PMC4721535

[iyac031-B56] Srikantha T , Lachke SA, Soll DR. Three mating type-like loci in *Candida glabrata*. Eukaryot Cell. 2003;2(2):328–340. doi:10.1128/EC.2.2.328-340.2003. 12684382 PMC154844

[iyac031-B57] Stamatakis A. RAxML-VI-HPC: maximum likelihood-based phylogenetic analyses with thousands of taxa and mixed models. Bioinformatics. 2006;22(21):2688–2690. doi:10.1093/bioinformatics/btl446. 16928733

[iyac031-B58] *Swofford DL. (n.d.) *PAUP: Phylogenetic Analysis Using Parsimony (and Other Methods) Version 4.0 beta (*2001*)*. Sinauer Associates. https://doi.org/10.1111/j.0014-3820.2002.tb00191.x

[iyac031-B59] Teunissen AW , Steensma HY. Review: the dominant flocculation genes of *Saccharomyces cerevisiae* constitute a new subtelomeric gene family. Yeast. 1995;11(11):1001–1013. doi:10.1002/yea.320111102. 7502576 10.1002/yea.320111102

[iyac031-B60] Turner SA , Butler G. The *Candida* pathogenic species complex. Cold Spring Harb Perspect Med. 2014;4(9):a019778. doi:10.1101/cshperspect.a019778. 25183855 PMC4143104

[iyac031-B61] Voss K , Auwera G.V D, Gentry J. Full-stack genomics pipelining with GATK4 + WDL + Cromwell’, in *18th Annual Bioinformatics Open Source Conference (BOSC 2017)*. *18th Annual Bioinformatics Open Source Conference (BOSC 2017)*. 2017. doi:10.7490/f1000research.1114634.1.

[iyac031-B62] Weig M , Jänsch L, Gross U, De Koster CG, Klis FM, De Groot PWJ. Systematic identification *in silico* of covalently bound cell wall proteins and analysis of protein-polysaccharide linkages of the human pathogen *Candida glabrata*. Microbiology (Reading). 2004;150(Pt 10):3129–3144. doi:10.1099/mic.0.27256-0. 15470094

[iyac031-B63] Wong S , Fares MA, Zimmermann W, Butler G, Wolfe KH. Evidence from comparative genomics for a complete sexual cycle in the “asexual” pathogenic yeast *Candida glabrata*. Genome Biol. 2003;4(2):R10.doi:10.1186/gb-2003-4-2-r10. 12620120 PMC151300

[iyac031-B64] Xu Z , Green B, Benoit N, Schatz M, Wheelan S, Cormack B. De novo genome assembly of *Candida glabrata* reveals cell wall protein complement and structure of dispersed tandem repeat arrays. Mol Microbiol. 2020;113(6):1209–1224. doi:10.1111/mmi.14488. 32068314

[iyac031-B65] Xu Z , Green B, Benoit N, Sobel JD, Schatz MC, Wheelan S, Cormack BP. Cell wall protein variation, break-induced replication, and subtelomere dynamics in *Candida glabrata*. Mol Microbiol. 2021;116(1):260–276. doi:10.1111/mmi.14707. 33713372

[iyac031-B66] Yang Z. PAML 4: phylogenetic analysis by maximum likelihood. Mol Biol Evol. 2007;24(8):1586–1591. doi:10.1093/molbev/msm088. 17483113

[iyac031-B67] Yang Z , Nielsen R. Estimating synonymous and nonsynonymous substitution rates under realistic evolutionary models. Mol Biol Evol. 2000;17(1):32–43. 10666704 10.1093/oxfordjournals.molbev.a026236

[iyac031-B68] Yapar N. Epidemiology and risk factors for invasive candidiasis. Clin Risk Manag. 2014;10:95–105. doi:10.2147/TCRM.S40160. PMC3928396 24611015

