# Supplementary material for: Population genetics and microevolution of clinical Candida glabrata reveals recombinant sequence types and hyper-variation within mitochondrial genomes, virulence genes, and drug targets
Source: Genetics. 2022 Feb 23;221(1):iyac031. doi: 10.1093/genetics/iyac031 (PMC9071574; doi:10.1093/genetics/iyac031)

A)

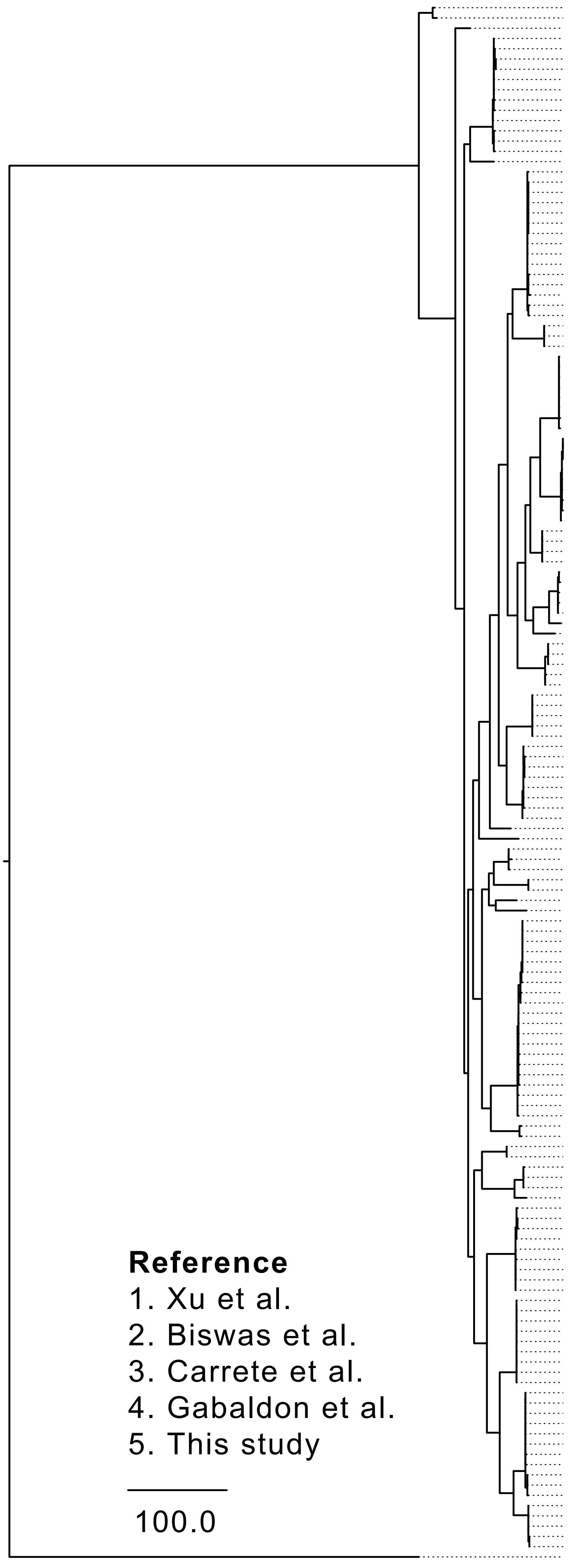


| | Clade | ST | Country | MAT | Reference |
|---|---|---|---|---|---|
| CBS138 | 5.3 | 15 | BE | ? | 1 |
| CG151 | 5.3 | 15 | GB | ? | 5 |
| CG57 | | 204 | GB | ? | 5 |
| M17 | 3 | 8 | US | a | 3 |
| M12 | 3 | 8 | US | alpha | 3 |
| WM18.64 | 3 | 8 | AU | ? | 2 |
| WM18.63 | 3 | 8 | AU | ? | 2 |
| CG82 | 3 | 8 | GB | ? | 5 |
| F2229 | 3 | 8 | FR | a | 3 |
| CST78 | 3 | 8 | US | a | 3 |
| I1718 | 3 | 8 | IT | a | 3 |
| F1019 | 3 | 8 | FR | a | 3 |
| CG52 | 3 | 8 | GB | ? | 5 |
| F1822 | 3 | 8 | FR | a | 3 |
| CG127 | 3 | 8 | GB | ? | 5 |
| WM18.45 | | 127 | AU | ? | 2 |
| CG66 | 5.1 | 10 | GB | ? | 5 |
| CG191B | 5.1 | 10 | GB | ? | 5 |
| CG191C | 5.1 | 10 | GB | ? | 5 |
| CG191F | 5.1 | 10 | GB | ? | 5 |
| CG191E | 5.1 | 10 | GB | ? | 5 |
| CG191A | 5.1 | 10 | GB | ? | 5 |
| CG191D | 5.1 | 10 | GB | ? | 5 |
| CG18A | 5.1 | 10 | GB | ? | 5 |
| CG18B | 5.1 | 10 | GB | ? | 5 |
| F15 | 5.1 | 10 | FR | a | 3 |
| CG134 | 5.1 | 10 | GB | ? | 5 |
| CG65 | 5.1 | 10 | GB | ? | 5 |
| WM18.26 | 5.1 | 10 | AU | ? | 2 |
| CG19 | 5.1 | 10 | GB | ? | 5 |
| CG76 | 5.1 | 10 | GB | ? | 5 |
| CG97C | | 25 | GB | ? | 5 |
| CG97A | | 25 | GB | ? | 5 |
| CG97B | | 25 | GB | ? | 5 |
| CG54 | 4 | 22 | GB | ? | 5 |
| EF0616Blo1 | 4 | 22 | FR | ? | 3 |
| CG55 | 4 | 22 | GB | ? | 5 |
| WM18.37 | 4 | 22 | AU | ? | 2 |
| EF1237Blo1 | 4 | 22 | FR | a | 3 |
| WM18.56 | 4 | 22 | AU | ? | 2 |
| EF1620Sto | 4 | 22 | FR | a | 3 |
| EI1815Blo1 | 4 | 22 | IT | alpha | 3 |
| CG79 | 5.2 | 6 | GB | ? | 5 |
| CG120 | 5.2 | 6 | GB | ? | 5 |
| CG150 | 5.2 | 6 | GB | ? | 5 |
| CG42 | 5.2 | 6 | GB | ? | 5 |
| F11 | 5.2 | 6 | FR | a | 3 |
| CG12 | 5.2 | 6 | GB | ? | 5 |
| CG121 | 5.2 | 6 | GB | ? | 5 |
| CG67 | 5.2 | 6 | GB | ? | 5 |
| WM18.38 | 5.2 | 6 | AU | ? | 2 |
| CG84F | | 67 | GB | ? | 5 |
| CG84H | | 67 | GB | ? | 5 |
| CG146 | | 67 | GB | ? | 5 |
| CG84G | | 67 | GB | ? | 5 |
| WM18.49 | | 26 | AU | ? | 2 |
| WM18.54 | | 26 | AU | ? | 2 |
| WM03.449 | | 26 | AU | ? | 2 |
| WM18.27 | | 26 | AU | ? | 2 |
| WM18.67 | | 26 | AU | ? | 2 |
| WM05.113 | | 18 | AU | ? | 2 |
| CG1 | | 177 | GB | ? | 5 |
| CG107A | | 36 | GB | ? | 5 |
| CG107B | | 36 | GB | ? | 5 |
| CG58 | | 36 | GB | ? | 5 |
| WM18.36 | | 36 | AU | ? | 2 |
| CG162 | | 36 | GB | ? | 5 |
| WM18.42 | | 123 | AU | ? | 2 |
| CG181 | | 123 | GB | ? | 5 |
| WM04.113 | | 123 | AU | ? | 2 |
| WM18.41 | | 123 | AU | ? | 2 |
| WM18.53 | | 123 | AU | ? | 2 |
| WM03.707 | | 83 | AU | ? | 2 |
| WM18.48 | | 83 | AU | ? | 2 |
| WM18.65 | | 83 | AU | ? | 2 |
| WM03.419 | | 83 | AU | ? | 2 |
| WM03.450 | | 83 | AU | ? | 2 |
| WM18.43 | | 83 | AU | ? | 2 |
| WM18.39 | | 83 | AU | ? | 2 |
| CG43 | | 83 | GB | ? | 5 |
| WM05.155 | | 126 | AU | ? | 2 |
| WM18.66 | | 124 | AU | ? | 2 |
| WM18.52 | | 16 | AU | ? | 2 |
| WM18.24 | | 16 | AU | ? | 2 |
| WM18.57 | | 16 | AU | ? | 2 |
| P35_2 | 6 | 136 | TW | a | 3 |
| P35_3 | 6 | 136 | TW | alpha | 3 |
| WM18.31 | | 45 | AU | ? | 2 |
| CG133 | | 147 | GB | ? | 5 |
| EG01004Sto | 7 | 3 | DE | a | 3 |
| F03013 | 7 | 3 | FR | a | 3 |
| WM18.59 | 7 | 3 | AU | ? | 2 |
| E1114 | 7 | 3 | BE | a | 3 |
| F15021 | 7 | 3 | FR | alpha | 3 |
| CG174 | 7 | 3 | GB | ? | 5 |
| WM18.44 | 7 | 3 | AU | ? | 2 |
| WM18.55 | 7 | 3 | AU | ? | 2 |
| WM18.30 | 7 | 3 | AU | ? | 2 |
| WM04.194 | 7 | 3 | AU | ? | 2 |
| CG167 | 7 | 3 | GB | ? | 5 |
| BG2 | 7 | 3 | US | a | 3 |
| CG69B | 7 | 3 | GB | ? | 5 |
| CG46 | 7 | 3 | GB | ? | 5 |
| WM18.35 | 7 | 3 | AU | ? | 2 |
| WM18.60 | 7 | 3 | AU | ? | 2 |
| CG203 | 7 | 3 | GB | ? | 5 |
| WM18.34 | 7 | 3 | AU | ? | 2 |
| M6 | 7 | 3 | US | a | 3 |
| CST110 | 7 | 3 | US | a | 3 |
| CG166 | | 24 | GB | ? | 5 |
| CG153 | | 24 | GB | ? | 5 |
| CG77 | | 4 | GB | ? | 5 |
| CG68A | | 4 | GB | ? | 5 |
| WM18.40 | | 46 | AU | ? | 2 |
| WM18.51 | | 46 | AU | ? | 2 |
| WM18.47 | | 46 | AU | ? | 2 |
| WM18.50 | | 59 | AU | ? | 2 |
| CG119 | 1 | 19 | GB | ? | 5 |
| M7 | 1 | 19 | US | alpha | 3 |
| CST80 | 1 | 19 | US | alpha | 3 |
| CST34 | 1 | 19 | US | alpha | 3 |
| BO101S | 1 | 19 | BE | alpha | 3 |
| EB101M | 1 | 19 | BE | alpha | 3 |
| B1012M | 1 | 19 | BE | alpha | 3 |
| CST109 | 1 | 19 | US | alpha | 3 |
| B1012S | 1 | 19 | BE | alpha | 3 |
| CG93H | | 162 | GB | ? | 5 |
| CG93K | | 162 | GB | ? | 5 |
| CG93E | | 162 | GB | ? | 5 |
| CG93B | | 162 | GB | ? | 5 |
| CG93J | | 162 | GB | ? | 5 |
| CG93I | | 162 | GB | ? | 5 |
| CG93D | | 162 | GB | ? | 5 |
| CG93A | | 162 | GB | ? | 5 |
| CG93C | | 162 | GB | ? | 5 |
| WM03.698 | 2 | 7 | AU | ? | 2 |
| WM18.33 | 2 | 7 | AU | ? | 2 |
| WM04.242 | 2 | 7 | AU | ? | 2 |
| CG48F | 2 | 7 | GB | ? | 5 |
| WM03.308 | 2 | 7 | AU | ? | 2 |
| CG48A | 2 | 7 | GB | ? | 5 |
| CG157 | 2 | 7 | GB | ? | 5 |
| CG78 | 2 | 7 | GB | ? | 5 |
| EB0911Sto | 2 | 7 | BE | alpha | 3 |
| CST35 | 2 | 7 | US | alpha | 3 |
| WM04.387 | 2 | 7 | AU | ? | 2 |
| CG53 | | 55 | GB | ? | 5 |
| WM05.111 | | 55 | AU | ? | 2 |
| WM18.62 | | 55 | AU | ? | 2 |
| CG124 | | 55 | GB | ? | 5 |
| CG144 | | 55 | GB | ? | 5 |
| *C. bracarensis* AGP | N/A | N/A | ? | ? | 4 |

B)

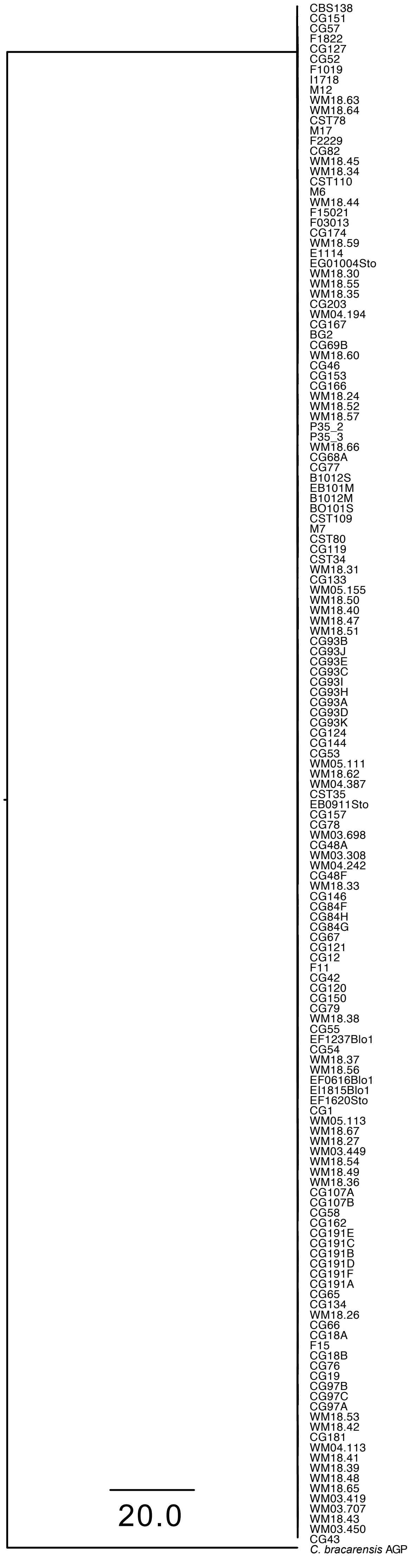

Supplement: iyac031_Supplementary_Figure_S1 [file iyac031_supplementary_figure_s1.pdf]
