# Supplementary figures and images for: Population genetics and microevolution of clinical Candida glabrata reveals recombinant sequence types and hyper-variation within mitochondrial genomes, virulence genes, and drug targets
Source: Genetics. 2022 Feb 23;221(1):iyac031. doi: 10.1093/genetics/iyac031 (PMC9071574; doi:10.1093/genetics/iyac031)

A)

10kb windows

| ST  | 4    | 6    | 7    | 8    | 10   | 15   | 16   | 18   | 19   | 22   | 24   | 25   | 26   | 36   | 45   | 46   | 55   | 59   | 67   | 83   | 123  | 124  | 126  | 127  | 136  | 147  | 162  | 177  | 204  |      |
|-----|------|------|------|------|------|------|------|------|------|------|------|------|------|------|------|------|------|------|------|------|------|------|------|------|------|------|------|------|------|------|
| 3   | 0.98 | 0.98 | 0.98 | 0.98 | 0.98 | 0.98 | 0.97 | 0.98 | 0.98 | 0.98 | 0.98 | 0.98 | 0.98 | 0.98 | 0.97 | 0.98 | 0.98 | 0.98 | 0.98 | 0.98 | 0.98 | 0.98 | 0.98 | 0.98 | 0.97 | 0.96 | 0.99 | 0.98 | 0.89 |      |
| 4   |      | 0.98 | 0.98 | 0.97 | 0.98 | 0.98 | 0.98 | 0.99 | 0.98 | 0.98 | 0.98 | 0.99 | 0.98 | 0.98 | 0.99 | 0.98 | 0.98 | 0.99 | 0.98 | 0.98 | 0.99 | 0.99 | 0.99 | 0.99 | 0.99 | 0.99 | 0.99 | 0.99 | 0.99 |      |
| 6   |      |      | 0.98 | 0.98 | 0.91 | 0.97 | 0.98 | 0.95 | 0.98 | 0.94 | 0.98 | 0.95 | 0.94 | 0.93 | 0.99 | 0.98 | 0.98 | 0.98 | 0.96 | 0.97 | 0.95 | 0.98 | 0.98 | 0.98 | 0.98 | 0.99 | 0.99 | 0.95 | 0.97 |      |
| 7   |      |      |      | 0.97 | 0.98 | 0.98 | 0.98 | 0.98 | 0.84 | 0.98 | 0.98 | 0.98 | 0.98 | 0.98 | 0.98 | 0.83 | 0.71 | 0.80 | 0.98 | 0.98 | 0.98 | 0.98 | 0.98 | 0.97 | 0.98 | 0.98 | 0.83 | 0.98 | 0.98 |      |
| 8   |      |      |      |      | 0.98 | 0.97 | 0.98 | 0.98 | 0.97 | 0.98 | 0.98 | 0.98 | 0.98 | 0.98 | 0.98 | 0.97 | 0.97 | 0.97 | 0.98 | 0.98 | 0.98 | 0.98 | 0.98 | 0.97 | 0.98 | 0.98 | 0.98 | 0.98 | 0.98 |      |
| 10  |      |      |      |      |      | 0.96 | 0.98 | 0.95 | 0.98 | 0.94 | 0.98 | 0.84 | 0.95 | 0.95 | 0.98 | 0.98 | 0.98 | 0.98 | 0.94 | 0.96 | 0.95 | 0.98 | 0.98 | 0.98 | 0.98 | 0.98 | 0.98 | 0.96 | 0.74 |      |
| 15  |      |      |      |      |      |      | 0.98 | 0.98 | 0.98 | 0.97 | 0.98 | 0.98 | 0.97 | 0.96 | 0.99 | 0.99 | 0.98 | 0.99 | 0.97 | 0.97 | 0.97 | 0.99 | 0.99 | 0.99 | 0.99 | 0.99 | 0.99 | 0.98 | 0.98 |      |
| 16  |      |      |      |      |      |      |      | 0.98 | 0.98 | 0.98 | 0.97 | 0.99 | 0.99 | 0.98 | 0.97 | 0.98 | 0.98 | 0.98 | 0.98 | 0.98 | 0.99 | 0.98 | 0.98 | 0.99 | 0.90 | 0.97 | 0.99 | 0.98 | 0.98 |      |
| 18  |      |      |      |      |      |      |      |      | 0.98 | 0.96 | 0.98 | 0.99 | 0.64 | 0.96 | 1.00 | 0.98 | 0.99 | 1.00 | 0.98 | 0.97 | 0.97 | 1.00 | 1.00 | 1.00 | 1.00 | 1.00 | 0.99 | 0.98 | 0.99 |      |
| 19  |      |      |      |      |      |      |      |      |      | 0.98 | 0.98 | 0.98 | 0.98 | 0.98 | 0.98 | 0.91 | 0.88 | 0.90 | 0.98 | 0.98 | 0.98 | 0.98 | 0.98 | 0.98 | 0.98 | 0.98 | 0.90 | 0.98 | 0.79 |      |
| 22  |      |      |      |      |      |      |      |      |      |      | 0.98 | 0.97 | 0.95 | 0.94 | 0.99 | 0.99 | 0.98 | 0.99 | 0.96 | 0.96 | 0.97 | 0.99 | 0.98 | 0.99 | 0.98 | 0.99 | 0.99 | 0.96 | 0.97 |      |
| 24  |      |      |      |      |      |      |      |      |      |      |      | 0.99 | 0.98 | 0.98 | 0.98 | 0.98 | 0.98 | 0.98 | 0.98 | 0.98 | 0.98 | 0.98 | 0.98 | 0.99 | 0.98 | 0.98 | 0.99 | 0.98 | 0.98 |      |
| 25  |      |      |      |      |      |      |      |      |      |      |      |      | 0.98 | 0.97 | 1.00 | 0.99 | 0.99 | 0.99 | 0.98 | 0.97 | 0.99 | 0.99 | 0.99 | 1.00 | 1.00 | 0.99 | 0.99 | 0.99 | 0.99 |      |
| 26  |      |      |      |      |      |      |      |      |      |      |      |      |      | 0.95 | 0.99 | 0.98 | 0.98 | 0.99 | 0.97 | 0.96 | 0.96 | 0.99 | 0.98 | 0.99 | 0.98 | 0.99 | 0.99 | 0.96 | 0.97 |      |
| 36  |      |      |      |      |      |      |      |      |      |      |      |      |      |      |      | 0.99 | 0.98 | 0.98 | 0.98 | 0.97 | 0.97 | 0.96 | 0.98 | 0.98 | 0.99 | 0.98 | 0.99 | 0.96 | 0.57 |      |
| 45  |      |      |      |      |      |      |      |      |      |      |      |      |      |      |      |      | 0.99 | 0.99 | 1.00 | 0.99 | 0.98 | 0.99 | 1.00 | 1.00 | 1.00 | 1.00 | 0.99 | 1.00 | 1.00 |      |
| 46  |      |      |      |      |      |      |      |      |      |      |      |      |      |      |      |      |      | 0.92 | 0.64 | 0.98 | 0.98 | 0.99 | 0.99 | 0.99 | 0.99 | 0.99 | 0.99 | 0.92 | 0.99 | 0.99 |
| 55  |      |      |      |      |      |      |      |      |      |      |      |      |      |      |      |      |      | 0.93 | 0.98 | 0.98 | 0.99 | 0.99 | 0.99 | 0.98 | 0.99 | 0.99 | 0.65 | 0.99 | 0.99 |      |
| 59  |      |      |      |      |      |      |      |      |      |      |      |      |      |      |      |      |      |      | 0.99 | 0.98 | 0.99 | 1.00 | 1.00 | 1.00 | 1.00 | 1.00 | 0.96 | 1.00 | 1.00 |      |
| 67  |      |      |      |      |      |      |      |      |      |      |      |      |      |      |      |      |      |      |      | 0.97 | 0.98 | 0.98 | 0.98 | 0.99 | 0.98 | 0.98 | 0.99 | 0.98 | 0.98 |      |
| 83  |      |      |      |      |      |      |      |      |      |      |      |      |      |      |      |      |      |      |      |      |      | 0.97 | 0.98 | 0.97 | 0.98 | 0.98 | 0.99 | 0.97 | 0.97 |      |
| 123 |      |      |      |      |      |      |      |      |      |      |      |      |      |      |      |      |      |      |      |      |      |      | 0.99 | 0.99 | 0.99 | 0.99 | 0.99 | 0.98 | 0.98 |      |
| 124 |      |      |      |      |      |      |      |      |      |      |      |      |      |      |      |      |      |      |      |      |      |      |      | 1.00 | 1.00 | 1.00 | 1.00 | 0.99 | 1.00 | 1.00 |
| 126 |      |      |      |      |      |      |      |      |      |      |      |      |      |      |      |      |      |      |      |      |      |      |      |      | 1.00 | 1.00 | 1.00 | 0.99 | 0.99 |      |
| 127 |      |      |      |      |      |      |      |      |      |      |      |      |      |      |      |      |      |      |      |      |      |      |      |      |      | 1.00 | 1.00 | 0.99 | 1.00 | 1.00 |
| 136 |      |      |      |      |      |      |      |      |      |      |      |      |      |      |      |      |      |      |      |      |      |      |      |      |      |      | 1.00 | 0.99 | 1.00 | 1.00 |
| 147 |      |      |      |      |      |      |      |      |      |      |      |      |      |      |      |      |      |      |      |      |      |      |      |      |      |      |      | 0.99 | 1.00 | 1.00 |
| 162 |      |      |      |      |      |      |      |      |      |      |      |      |      |      |      |      |      |      |      |      |      |      |      |      |      |      |      |      | 0.99 | 0.99 |
| 177 |      |      |      |      |      |      |      |      |      |      |      |      |      |      |      |      |      |      |      |      |      |      |      |      |      |      |      |      |      | 0.99 |

B)

5kb windows

| ST  | 4    | 6    | 7    | 8    | 10   | 15   | 16   | 18   | 19   | 22   | 24   | 25   | 26   | 36   | 45   | 46   | 55   | 59   | 67   | 83   | 123  | 124  | 126  | 127  | 136  | 147  | 162  | 177  | 204  |      |
|-----|------|------|------|------|------|------|------|------|------|------|------|------|------|------|------|------|------|------|------|------|------|------|------|------|------|------|------|------|------|------|
| 3   | 0.90 | 0.91 | 0.92 | 0.91 | 0.85 | 0.88 | 0.86 | 0.88 | 0.85 | 0.90 | 0.87 | 0.89 | 0.89 | 0.81 | 0.82 | 0.90 | 0.90 | 0.89 | 0.89 | 0.90 | 0.90 | 0.88 | 0.88 | 0.89 | 0.85 | 0.81 | 0.92 | 0.88 | 0.88 |      |
| 4   |      | 0.98 | 0.97 | 0.96 | 0.85 | 0.98 | 0.98 | 0.99 | 0.77 | 0.98 | 0.98 | 0.99 | 0.98 | 0.72 | 0.99 | 0.98 | 0.98 | 0.99 | 0.98 | 0.98 | 0.98 | 0.99 | 0.99 | 1.00 | 0.99 | 0.99 | 0.99 | 0.99 | 0.99 |      |
| 6   |      |      | 0.97 | 0.97 | 0.74 | 0.95 | 0.98 | 0.93 | 0.86 | 0.91 | 0.97 | 0.93 | 0.91 | 0.72 | 0.98 | 0.98 | 0.98 | 0.98 | 0.94 | 0.95 | 0.93 | 0.98 | 0.97 | 0.98 | 0.98 | 0.98 | 0.98 | 0.91 | 0.95 |      |
| 7   |      |      |      | 0.97 | 0.89 | 0.97 | 0.97 | 0.97 | 0.68 | 0.97 | 0.98 | 0.98 | 0.97 | 0.84 | 0.98 | 0.81 | 0.72 | 0.78 | 0.97 | 0.97 | 0.98 | 0.98 | 0.98 | 0.96 | 0.98 | 0.98 | 0.83 | 0.97 | 0.97 |      |
| 8   |      |      |      |      | 0.89 | 0.97 | 0.98 | 0.97 | 0.84 | 0.97 | 0.98 | 0.98 | 0.97 | 0.85 | 0.98 | 0.97 | 0.96 | 0.97 | 0.97 | 0.97 | 0.97 | 0.97 | 0.97 | 0.96 | 0.98 | 0.98 | 0.97 | 0.97 | 0.97 |      |
| 10  |      |      |      |      |      | 0.75 | 0.86 | 0.72 | 0.80 | 0.77 | 0.82 | 0.57 | 0.76 | 0.66 | 0.85 | 0.86 | 0.87 | 0.84 | 0.75 | 0.81 | 0.76 | 0.84 | 0.81 | 0.84 | 0.84 | 0.85 | 0.88 | 0.71 | 0.71 |      |
| 15  |      |      |      |      |      |      | 0.98 | 0.99 | 0.80 | 0.96 | 0.97 | 0.99 | 0.95 | 0.60 | 0.99 | 0.99 | 0.98 | 0.99 | 0.96 | 0.96 | 0.95 | 0.99 | 0.99 | 0.99 | 0.98 | 0.99 | 0.99 | 0.99 | 0.98 |      |
| 16  |      |      |      |      |      |      |      | 0.98 | 0.83 | 0.98 | 0.97 | 0.98 | 0.98 | 0.74 | 0.96 | 0.98 | 0.98 | 0.98 | 0.98 | 0.98 | 0.98 | 0.98 | 0.98 | 0.98 | 0.97 | 0.96 | 0.99 | 0.98 | 0.98 |      |
| 18  |      |      |      |      |      |      |      |      | 0.77 | 0.94 | 0.98 | 0.99 | 0.71 | 0.54 | 1.00 | 0.98 | 0.98 | 0.99 | 0.97 | 0.96 | 0.97 | 1.00 | 0.99 | 0.99 | 1.00 | 1.00 | 0.99 | 0.95 | 0.96 |      |
| 19  |      |      |      |      |      |      |      |      |      | 0.86 | 0.81 | 0.82 | 0.83 | 0.64 | 0.80 | 0.69 | 0.66 | 0.62 | 0.82 | 0.85 | 0.83 | 0.79 | 0.77 | 0.73 | 0.81 | 0.80 | 0.71 | 0.78 | 0.78 |      |
| 22  |      |      |      |      |      |      |      |      |      |      | 0.98 | 0.96 | 0.94 | 0.71 | 0.98 | 0.98 | 0.98 | 0.98 | 0.93 | 0.95 | 0.96 | 0.98 | 0.97 | 0.98 | 0.98 | 0.98 | 0.98 | 0.92 | 0.96 |      |
| 24  |      |      |      |      |      |      |      |      |      |      |      | 0.98 | 0.97 | 0.67 | 0.98 | 0.98 | 0.98 | 0.98 | 0.97 | 0.97 | 0.98 | 0.98 | 0.98 | 0.98 | 0.98 | 0.98 | 0.99 | 0.98 | 0.98 |      |
| 25  |      |      |      |      |      |      |      |      |      |      |      |      | 0.99 | 0.64 | 0.99 | 0.99 | 0.99 | 0.99 | 0.98 | 0.96 | 1.00 | 0.99 | 0.99 | 0.99 | 1.00 | 0.99 | 0.99 | 0.99 | 0.98 |      |
| 26  |      |      |      |      |      |      |      |      |      |      |      |      |      | 0.68 | 0.99 | 0.99 | 0.98 | 0.99 | 0.96 | 0.96 | 0.94 | 0.99 | 0.98 | 0.98 | 0.98 | 0.99 | 0.98 | 0.96 | 0.96 |      |
| 36  |      |      |      |      |      |      |      |      |      |      |      |      |      |      | 0.67 | 0.74 | 0.77 | 0.66 | 0.68 | 0.77 | 0.69 | 0.66 | 0.62 | 0.66 | 0.70 | 0.67 | 0.83 | N/A  | 0.55 |      |
| 45  |      |      |      |      |      |      |      |      |      |      |      |      |      |      |      | 0.99 | 0.99 | 0.99 | 0.98 | 0.98 | 0.99 | 1.00 | 0.99 | 0.99 | 1.00 | 0.97 | 0.99 | 0.99 | 0.99 |      |
| 46  |      |      |      |      |      |      |      |      |      |      |      |      |      |      |      |      | 0.97 | 0.71 | 0.98 | 0.98 | 0.99 | 0.99 | 0.99 | 0.98 | 0.99 | 0.99 | 0.89 | 0.98 | 0.99 |      |
| 55  |      |      |      |      |      |      |      |      |      |      |      |      |      |      |      |      |      | 0.94 | 0.98 | 0.98 | 0.98 | 0.98 | 0.98 | 0.98 | 0.98 | 0.99 | 0.58 | 0.98 | 0.98 |      |
| 59  |      |      |      |      |      |      |      |      |      |      |      |      |      |      |      |      |      |      | 0.98 | 0.98 | 0.99 | 1.00 | 1.00 | 0.99 | 1.00 | 1.00 | 0.94 | 0.99 | 0.99 |      |
| 67  |      |      |      |      |      |      |      |      |      |      |      |      |      |      |      |      |      |      |      | 0.96 | 0.97 | 0.98 | 0.98 | 0.98 | 0.98 | 0.98 | 0.98 | 0.97 | 0.97 |      |
| 83  |      |      |      |      |      |      |      |      |      |      |      |      |      |      |      |      |      |      |      |      |      | 0.96 | 0.98 | 0.96 | 0.98 | 0.98 | 0.98 | 0.96 | 0.95 |      |
| 123 |      |      |      |      |      |      |      |      |      |      |      |      |      |      |      |      |      |      |      |      |      |      | 0.99 | 0.99 | 0.99 | 0.98 | 0.99 | 0.99 | 0.97 | 0.97 |
| 124 |      |      |      |      |      |      |      |      |      |      |      |      |      |      |      |      |      |      |      |      |      |      |      | 0.99 | 0.99 | 1.00 | 1.00 | 0.99 | 0.99 | 1.00 |
| 126 |      |      |      |      |      |      |      |      |      |      |      |      |      |      |      |      |      |      |      |      |      |      |      |      | 0.99 | 1.00 | 1.00 | 0.99 | 0.99 | 0.99 |
| 127 |      |      |      |      |      |      |      |      |      |      |      |      |      |      |      |      |      |      |      |      |      |      |      |      |      | 1.00 | 1.00 | 0.98 | 0.99 | 0.99 |
| 136 |      |      |      |      |      |      |      |      |      |      |      |      |      |      |      |      |      |      |      |      |      |      |      |      |      |      | 1.00 | 0.99 | 1.00 | 1.00 |
| 147 |      |      |      |      |      |      |      |      |      |      |      |      |      |      |      |      |      |      |      |      |      |      |      |      |      |      |      | 0.99 | 0.99 | 1.00 |
| 162 |      |      |      |      |      |      |      |      |      |      |      |      |      |      |      |      |      |      |      |      |      |      |      |      |      |      |      |      | 0.99 | 0.99 |
| 177 |      |      |      |      |      |      |      |      |      |      |      |      |      |      |      |      |      |      |      |      |      |      |      |      |      |      |      |      |      | 0.98 |

FST

0.9-1

0.8-0.9

0.7-0.8

0.6-0.7

0.5-0.6

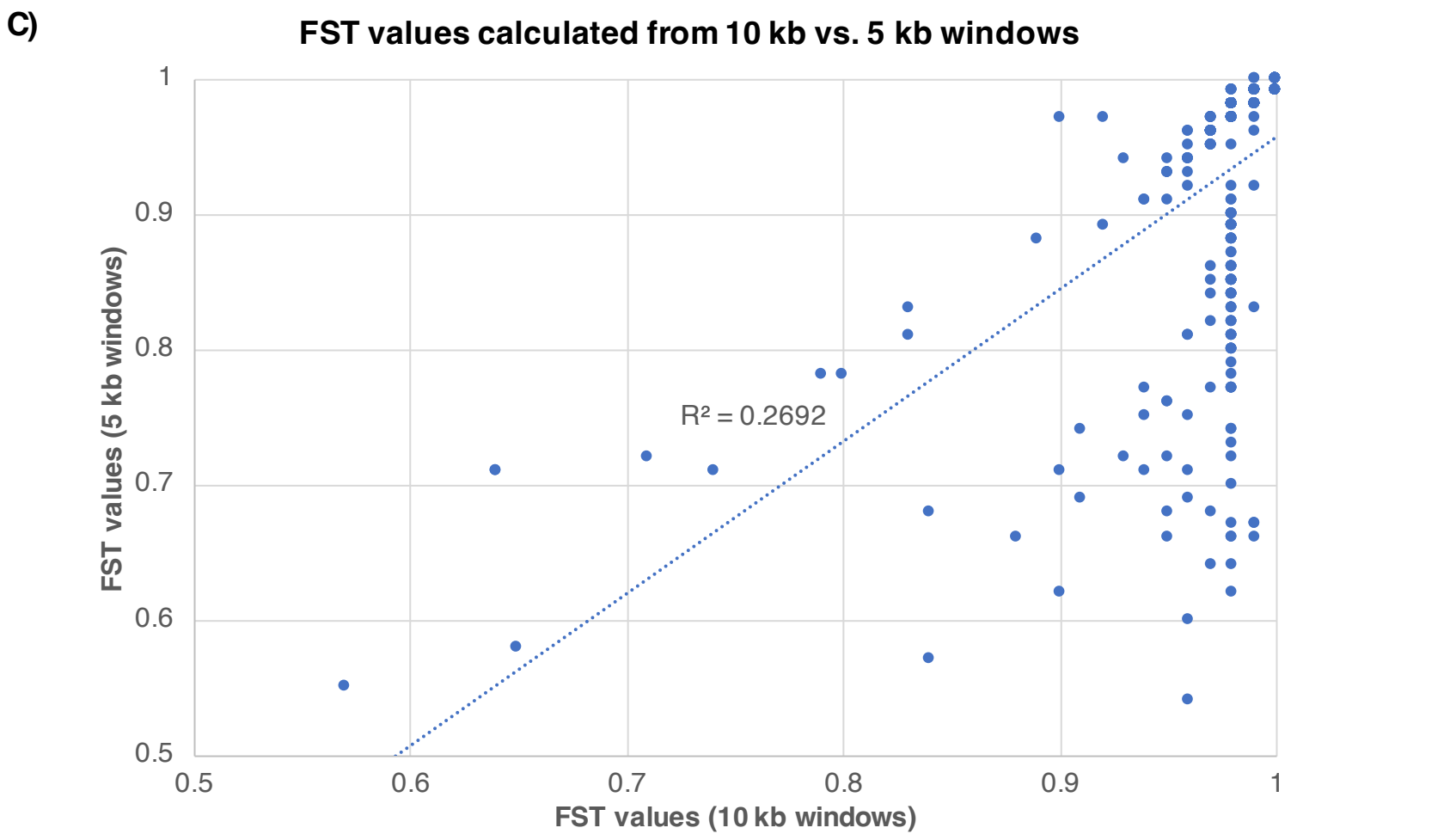

Supplement: iyac031_Supplementary_Figure_S2 [file iyac031_supplementary_figure_s2.pdf]

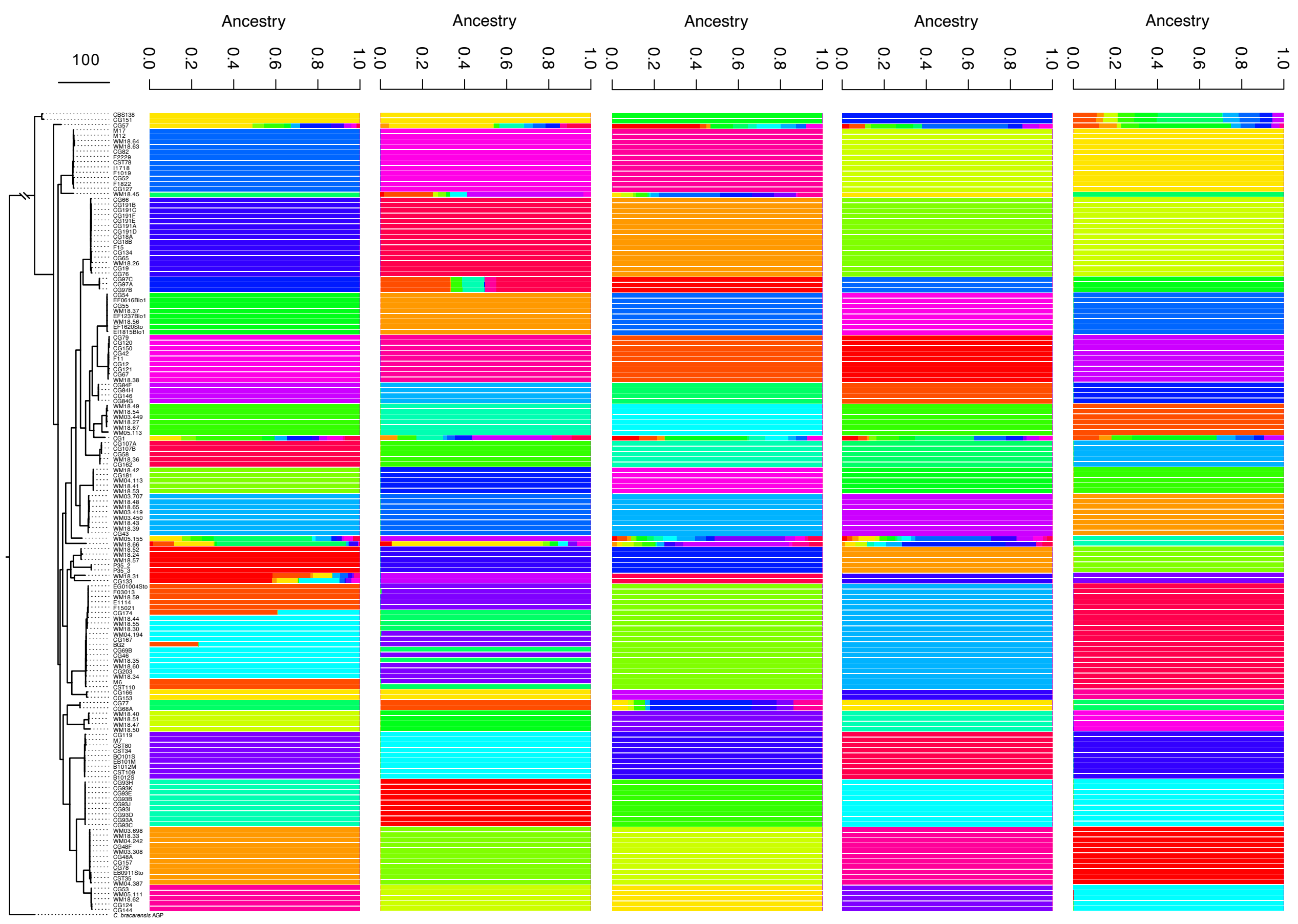

Supplement: iyac031_Supplementary_Figure_S4 [file iyac031_supplementary_figure_s4.pdf]

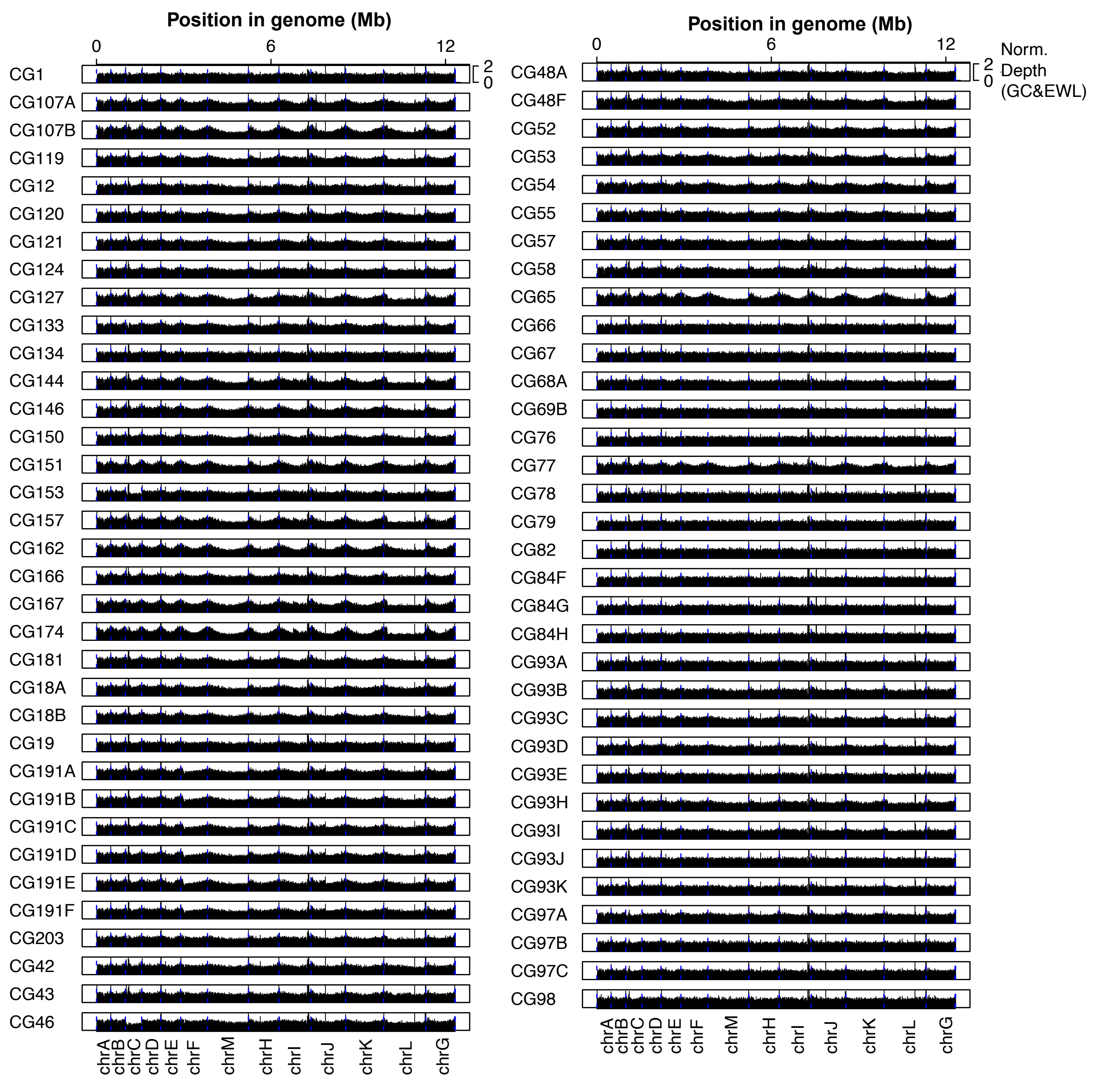

Supplement: iyac031_Supplementary_Figure_S5 [file iyac031_supplementary_figure_s5.pdf]
