# Supplementary material for: Population genetics and microevolution of clinical Candida glabrata reveals recombinant sequence types and hyper-variation within mitochondrial genomes, virulence genes, and drug targets
Source: Genetics. 2022 Feb 23;221(1):iyac031. doi: 10.1093/genetics/iyac031 (PMC9071574; doi:10.1093/genetics/iyac031)
Supplement: iyac031_Supplementary_Figure_S3 [file iyac031_supplementary_figure_s3.pdf]

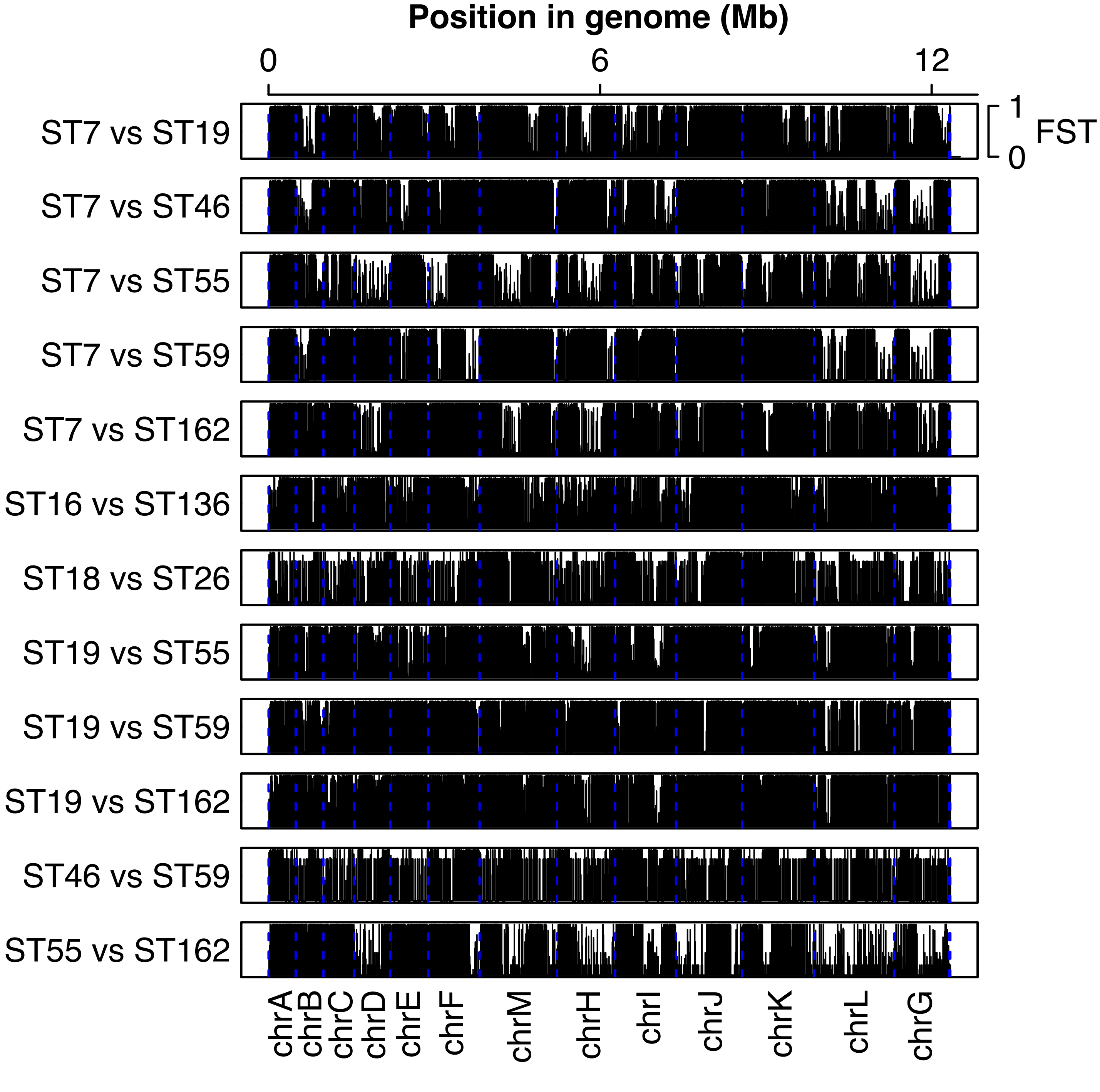

Position in genome (Mb)
0
6
12
1
FST
0
ST7 vs ST19
ST7 vs ST46
ST7 vs ST55
ST7 vs ST59
ST7 vs ST162
ST16 vs ST136
ST18 vs ST26
ST19 vs ST55
ST19 vs ST59
ST19 vs ST162
ST46 vs ST59
ST55 vs ST162
chrA
chrB
chrC
chrD
chrE
chrF
chrM
chrH
chrI
chrJ
chrK
chrL
chrG
