# Supplementary material for: Population genetics and microevolution of clinical Candida glabrata reveals recombinant sequence types and hyper-variation within mitochondrial genomes, virulence genes, and drug targets
Source: Genetics. 2022 Feb 23;221(1):iyac031. doi: 10.1093/genetics/iyac031 (PMC9071574; doi:10.1093/genetics/iyac031)
Supplement: iyac031_Supplemental_Material_Legends [file iyac031_supplemental_material_legends.docx]

Supplementary material for:

Population genetics and microevolution of clinical *Candida glabrata* reveals recombinant sequence types and hyper-variation within mitochondrial genomes, virulence genes and drug-targets

Authors: Nicolas Helmstetter^*,††^, Aleksandra D. Chybowska^†,††^, Christopher Delaney^‡^, Alessandra Da Silva Dantas^*^, Hugh Gifford^*^, Theresa Wacker^*^, Carol Munro^†^, Adilia Warris^*^, Brian Jones^§^, Christina A. Cuomo^**^, Duncan Wilson^*^, Gordon Ramage^‡^ and Rhys A. Farrer^*,**^

Authors institutional affiliations:

^*^Medical Research Council Centre for Medical Mycology at the University of Exeter, Exeter, UK, EX4 4QD.

^†^Institute of Medical Sciences, University of Aberdeen, Aberdeen, UK, AB25 2ZD.

^‡^School of Medicine, College of Medical, Veterinary and Life Sciences, University of Glasgow, Glasgow, UK. G12 8QQ.

^§^Institute of Infection, Immunity & Inflammation, University of Glasgow, UK. G12 8TA.

^**^Broad Institute of MIT and Harvard, Cambridge, Massachusetts, USA. 02142.

^††^These authors contributed equally.

Corresponding author: Rhys A. Farrer. MRC Centre for Medical Mycology

University of Exeter, Geoffrey Pope Building, Stocker Road, Exeter EX4 4QD, UK. T: +44(0)1392 727594; E: r.farrer@exeter.ac.uk

Supplemental Figure Legends

Figure S1. Phylogenetic trees of *C. glabrata*. All genomic sites that were either a homozygous reference or SNP in every isolate of *C. glabrata* and *C. bracarensis* AGP (Correia *et al.*, 2006) for rooting were identified using ECATools and concatenated into a FASTA file. A) A neighbor-joining tree constructed with PAUP. Scale bar indicates the distance based on substitutions per site. B) A maximum likelihood tree constructed using RAxML PThreads v.7.7.8 (Stamatakis, 2006) using the general-time-reversible model and CAT rate approximation with 100 bootstrap support. Branch lengths indicate the mean number of changes per site. The clade according to Carreté L *et al.* 2018 (Carreté *et al.*, 2018), as well as sequence type (ST), country code (AU = Australia, BE = Belgium, DE = Germany, FR = France, GB = Great Britain, IT = Italy, TW = Taiwan, US = United States), MAT and reference are also shown.

Figure S2. Mean F_ST_ values from pairwise comparisons of each sequence type (ST) calculated from a) 10 kb non-overlapping windows and b) 5 kb windows. C) Mean F_ST_ values from 10 kb windows were similar to values calculated from 5 kb windows, with a mean difference of -0.046 per pairwise comparison.

Figure S3. Non-overlapping 10 kb windows showing *F_ST_* values for 12 pairwise comparisons that had long genomic regions with lower values.

Figure S4. Five independent runs of ADMIXTURE using K = 20 and time-based seed values, revealing several isolates with evidence of mixed ancestry. Isolates are ordered according to the neighbor-joining tree constructed with PAUP in Figure S1.

Figure S5. Non-overlapping 10 kb windows showing normalized depth of coverage (including GC normalization by percentiles; GC, and excluding ambiguous sites (effective window length)).

Figure S6. Integrated Genome Viewer (IGV) screenshots for the reference isolate CBS138, and all isolates compared in this study, indicating substantial differences between our Illumina sequences and the mitochondrial assembly. Gene features are shown as a track (directionality indicated by arrows), and the read alignment from the BAM files are shown for each isolate, where peaks indicate higher depth, and colors on the peaks indicate discrepancies to the reference base: green = A, blue = C, red = T, brown = G, purple = insertion)

Supplemental Table Legends

Table S1. Metadata for clinical Scottish *C. glabrata* sequenced and analysed in this study.

Table S2. Haploid variant call summary based on alignments to the published nuclear and mitochondrial assembly of ST15 CBS138. These variants form the basis for the population genetic and comparative genomics tests.

Table S3. Diploid variant call summary. Diploid variant calls were used to check for any evidence of heterozygosity suggestive of diploidy. All heterozygous sites (single nucleotide heterozygous positions + heterozygous insertions + heterozygous deletions) amounted to <0.0404% (0.4 per Kb) of total positions called per isolate, suggesting these were errors and not evidence of diploidy.

Table S4. Counts of presence/absence (P/A) polymorphisms in each isolate, based on zero reads aligning to the ST15 CBS138 nuclear and mitochondrial genomic regions encoding gene sequences.

Table S5. A summary of *dN*/*dS* (ω) and nonsense mutations found across every gene in isolates representing each of the sequence types (ST).

Table S6. Details of 21 genes (found 67 times across all STs) with *dN*/*dS* (ω) > 1, which belonged to our set of 129 “genes of interest” including adhesions (e.g. EPA genes), aspartic proteases, phospholipases, cell wall biogenesis, structural wall proteins, regulatory, efflux pumps (all genes in Table 1 of (Weig *et al.*, 2004)), as well as both FKS and 12 ERG pathway genes.

Table S7. Counts of all microevolutionary changes documented between seven sets of between 2 and 9 *C. glabrata* isolates from recurrent cases of candidiasis.

Table S8. Details of 29 genes that had microevolutionary changes documented between seven sets of between 2 and 9 *C. glabrata* isolates from recurrent cases of candidiasis, and belonged to our set of 129 “genes of interest” including adhesions (e.g. EPA genes), aspartic proteases, phospholipases, cell wall biogenesis, structural wall proteins, regulatory, efflux pumps (all genes in Table 1 of (Weig *et al.*, 2004)), as well as both FKS and 12 ERG pathway genes. For each mutation, the information is encoded in a string with details separated by a semi colon. The first detail in variant type (e.g. ref_to_snp, where ref=reference), the second is location in CDS by nucleotide count, the third is the codon position (1, 2 or 3), the fourth is codon found along with amino acid position and type. Finally, a short description is given e.g. INSERTION and DELETION along with the number of nucleotides, or SYN=Synonymous, NSY=Non-synonymous, and NON=Nonsense.

**References**

Carreté, L. *et al.* (2018) ‘Patterns of genomic variation in the opportunistic pathogen *Candida glabrata* suggest the existence of mating and a secondary association with humans’, *Current biology: CB*, 28(1), pp. 15-27.e7. doi:10.1016/j.cub.2017.11.027.

Correia, A. *et al.* (2006) ‘*Candida bracarensis sp. nov.*, a novel anamorphic yeast species phenotypically similar to *Candida glabrata*’, *International Journal of Systematic and Evolutionary Microbiology*, 56(Pt 1), pp. 313–317. doi:10.1099/ijs.0.64076-0.

Stamatakis, A. (2006) ‘RAxML-VI-HPC: maximum likelihood-based phylogenetic analyses with thousands of taxa and mixed models’, *Bioinformatics*, 22(21), pp. 2688–2690. doi:10.1093/bioinformatics/btl446.

Weig, M. *et al.* (2004) ‘Systematic identification *in silico* of covalently bound cell wall proteins and analysis of protein-polysaccharide linkages of the human pathogen *Candida glabrata*’, *Microbiology (Reading, England)*, 150(Pt 10), pp. 3129–3144. doi:10.1099/mic.0.27256-0.
